# Applying Neural Networks to Analyse Inflammatory, Sociodemographic, and Psychological Factors in Non-Melanoma Skin Cancer and Colon Cancer: A Statistical and Artificial Intelligence Approach

**DOI:** 10.3390/diagnostics14232759

**Published:** 2024-12-07

**Authors:** Răzvan Mercuţ, Marius Eugen Ciurea, Emil Tiberius Traşcă, Mihaela Ionescu, Maria Filoftea Mercuţ, Patricia Mihaela Rădulescu, Cristina Călăraşu, Liliana Streba, Alin Gabriel Ionescu, Dumitru Rădulescu

**Affiliations:** 1 Department of Plastic and Reconstructive Surgery, University of Medicine and Pharmacy of Craiova, 200349 Craiova, Romania; razvan.mercut@umfcv.ro (R.M.); marius.ciurea@umfcv.ro (M.E.C.); 2 The Surgery Clinic of “Dr. Ștefan Odobleja Emergency Military Hospital”, General Surgery Department, University of Medicine and Pharmacy of Craiova, 200349 Craiova, Romania; emil.trasca@umfcv.ro (E.T.T.); dr_radulescu_dumitru@yahoo.com (D.R.); 3 Department of Medical Informatics and Biostatistics, University of Medicine and Pharmacy of Craiova, 200349 Craiova, Romania; 4 Department of Ophthalmology, University of Medicine and Pharmacy of Craiova, 200349 Craiova, Romania; 5 Department of Pneumology, University of Pharmacy and Medicine Craiova, 200349 Craiova, Romania; paty_miha@yahoo.com (P.M.R.); calarasu.cristina@yahoo.com (C.C.); 6 Department of Oncology, University of Medicine and Pharmacy of Craiova, 200349 Craiova, Romania; liliana.streba@umfcv.ro; 7 Department of Medical History, University of Pharmacy and Medicine Craiova, 200349 Craiova, Romania; alin.ionescu@umfcv.ro

**Keywords:** non-melanoma skin cancer, colorectal cancer, inflammatory factors, psychological factors, neural networks

## Abstract

**Background/Objectives:** Chronic inflammation and psychosocial factors significantly influence cancer progression and patient behavior in seeking medical care. Understanding their interplay is essential for enhancing early detection and developing personalized treatment strategies. This study aims to develop a comprehensive patient profiling model by comparing non-melanoma skin cancer (NMSC) and colorectal cancer (CRC). The goal is to identify common and distinct patterns in inflammation and psychosocial factors that affect disease progression and clinical presentation. **Methods:** We conducted a comparative analysis of patients diagnosed with NMSC and CRC, integrating clinical data with sociodemographic and psychological assessments. Advanced neural network algorithms were employed to detect subtle patterns and interactions among these factors. Based on the analysis, a cancer risk assessment questionnaire was developed to stratify patients into low-, moderate-, and high-risk categories. **Results:** Patients with low systemic inflammation and adequate vagal tone, supported by a stable family environment, demonstrated heightened sensitivity to subclinical symptoms, enabling earlier diagnosis and timely intervention. Conversely, patients with high systemic inflammation and reduced vagal tone, often influenced by chronic stress and unstable family environments, presented at more advanced disease stages. The developed risk assessment tool effectively classified patients into distinct risk categories, facilitating targeted preventive measures and personalized therapeutic strategies. Neural network profiling revealed significant interactions between biological and psychosocial factors, enhancing our understanding of their combined impact on cancer progression. **Conclusions:** The integrated profiling approach and the newly developed risk assessment questionnaire have the potential to transform cancer management by improving early detection, personalizing treatment strategies, and addressing psychosocial factors. This model not only enhances clinical outcomes and patient quality of life but also offers a framework adaptable to other cancer types, promoting a holistic and patient-centered approach in oncology.

## 1. Introduction

Cancer remains one of the most pressing global public health challenges, standing as the second leading cause of death in the United States and posing a growing worldwide concern. The 2023 cancer report estimates nearly 1,958,310 new cancer cases and 609,820 cancer-related deaths in the United States alone, while the most recent data from 2020 in Europe project approximately 4 million new cases and 1.9 million cancer-related deaths [1,2]. The COVID-19 pandemic significantly impacted the diagnosis and management of acute medical conditions, including cancer, leading to a temporary decrease in reported cases. However, this decrease is anticipated to yield a delayed increase in advanced-stage diagnoses and related mortality rates due to the interruptions in regular screening and early detection services [3,4,5,6]. In 2022, an estimated 19.3 million new cancer cases and 10 million deaths worldwide underscored the disease’s severity and scope [7,8]. While breast cancer remains the most frequently diagnosed, colorectal cancer (CRC) and non-melanoma skin cancer (NMSC) exhibit notable incidence rates, with colorectal cancer particularly linked to high mortality [9,10].

Colorectal cancer, increasingly prevalent in both developed and middle-income nations, is linked to risk factors that include urbanization and Western lifestyle behaviors such as a high-fat, low-fiber diet, physical inactivity, and alcohol consumption [11,12]. In 2020 alone, the global incidence of CRC was estimated at 1.93 million new cases, reflecting an upward trend, especially among men in Western Europe and North America [9]. Similarly, non-melanoma skin cancer is predominantly influenced by ultraviolet (UV) radiation exposure, the primary risk factor for NMSC. Outdoor professions significantly increase the risk of NMSC due to chronic UV exposure, with skin type and genetic susceptibility further influencing disease risk, particularly among immunosuppressed individuals [13,14]. Foundational studies, such as Lear et al. (1998) [15], have established the link between fair skin and increased skin cancer risk, which has since been corroborated by subsequent research. Both CRC and NMSC risks are modulated by genetic and environmental factors, with certain gene polymorphisms, like those in GST, being associated with increased vulnerability in high-UV settings for NMSC [16]. Family history, as highlighted in recent reviews [17], also plays a significant role in CRC risk.

Sociodemographic factors such as age, gender, socioeconomic status (SES), and geographic location also contribute to cancer outcomes. Age and gender are critical in shaping cancer incidence and survival, with cancer rates rising with age and generally lower survival rates among men [18,19]. SES significantly influences cancer prognosis, as patients with lower SES are often diagnosed at later stages and experience reduced access to treatment, leading to poorer outcomes [20]. Urban and rural residents further impact cancer outcomes; urban patients generally benefit from earlier diagnoses and improved survival due to access to advanced healthcare services, while rural residents may encounter barriers that delay diagnosis and treatment [21,22,23]. Psychological factors, including stress, anxiety, and depression, also play a pivotal role in cancer progression, influencing biological pathways such as angiogenesis and tumor invasiveness, which accelerate disease progression. Additionally, social support has been shown to ameliorate the adverse effects of psychological stress, improving quality of life and survival rates in patients with cancer [24,25,26,27,28].

Machine learning (ML) methodologies are increasingly applied in oncology, complementing traditional statistical methods by providing sophisticated, predictive models for cancer diagnosis and prognosis. Algorithms such as artificial neural networks (ANNs), support vector machines (SVMs), and decision trees (DTs) are commonly used to classify patients based on cancer progression risk, enhancing the accuracy of clinical decision-making. While foundational studies have demonstrated the statistical importance of these approaches [29,30], more recent work has focused on integrating these methods into practical machine learning applications [31]. Nevertheless, ML’s effectiveness hinges on its integration with conventional statistical approaches (e.g., t-tests, ANOVA, regression analysis), which facilitate validation and interpretation of ML-derived results [32,33]. This combined approach allows for greater thoroughness and interpretability in clinical data analysis, as traditional statistical methods validate the reliability of complex ML models [34]. The recent literature highlights the effectiveness of this hybrid approach, demonstrating how ML algorithms can uncover nuanced patterns that classical methods may miss, ultimately optimizing clinical decision-making in oncology [35].

### 1.1. Summary of Key Studies in the Literature

The table below presents a summary of relevant studies, highlighting their contributions to understanding cancer incidence, progression, and the factors that influence these outcomes (Table 1). 

The studies underscore the roles of sociodemographic, environmental, psychological, and biological factors, as well as the methodologies used to investigate these relationships.

### 1.2. Study Objectives and Significance

Given the complexity and variability of the factors involved in cancer development and progression, adopting an integrated approach to profiling patients with non-melanoma skin cancer and colorectal cancer is essential. Recent studies emphasize the importance of combining traditional data (clinical and demographic) with advanced machine learning techniques to develop accurate and personalized predictive models [39,40,41]. These approaches not only improve diagnostic and prognostic accuracy but also contribute to the personalization of treatment, which can lead to increased survival rates and improved quality of life for patients [37,38].

The motivation behind this research stems from the increasing evidence that both biological and psychosocial factors play a critical role in cancer progression. Although previous studies have investigated these aspects separately, there is a need for an integrated approach that examines how systemic inflammation, stress, and socio-demographic conditions interact to influence disease outcomes. This study aims to bridge this gap by utilizing a hybrid methodology that combines conventional statistical techniques with machine learning models to identify patient profiles based on these variables. The primary focus is on understanding how different factors contribute to early detection and progression in non-melanoma skin cancer and colorectal cancer. Through this approach, we aim to offer more personalized treatment strategies and improve patient outcomes. The research was conducted in several stages: data collection from clinical sources, statistical analysis to identify significant variables, and the application of neural networks to develop predictive profiles for cancer progression.

### 1.3. Justification for Comparing NMSC and CRC

The comparison between non-melanoma skin cancer (NMSC) and colorectal cancer (CRC) in this study is grounded in several critical factors that enhance our understanding of cancer progression and inform therapeutic strategies.

Firstly, both NMSC and CRC are prevalent malignancies that impose substantial burdens on healthcare systems worldwide. NMSC is the most common cancer among light-skinned populations, with incidence rates escalating due to increased ultraviolet (UV) radiation exposure and aging demographics [42]. CRC is similarly significant, ranking among the leading causes of cancer-related mortality globally, with over 1.2 million new cases annually [43].

Secondly, these cancers exhibit contrasting clinical presentations concerning lesion visibility and symptomatology, which profoundly influence patient behavior in seeking medical care. NMSC presents with visible skin lesions that are readily noticeable, often prompting earlier medical consultation [44]. In contrast, CRC involves internal lesions with nonspecific or absent early-stage symptoms, frequently leading to delayed diagnosis [45]. By examining both cancer types, our study explores whether lesion visibility is a determinant in early detection and how subtle symptoms, potentially influenced by inflammatory processes or psychological stress, affect patient engagement with healthcare services.

Thirdly, chronic inflammation serves as a common pathogenic mechanism in both NMSC and CRC, activating signaling pathways such as NF-κB and STAT3 that promote cellular proliferation, angiogenesis, and immune evasion [46]. In NMSC, repeated UV exposure induces DNA damage and local inflammatory responses, initiating carcinogenesis [47]. In CRC, chronic intestinal inflammation, as seen in inflammatory bowel diseases, is a major driver of tumor development [48]. Comparative analysis of these mechanisms may reveal pertinent similarities and differences essential for advancing therapeutic interventions.

Moreover, sociodemographic and psychological factors—including socioeconomic status, age, and residential environment—significantly influence the incidence and survival rates of both cancers. Chronic stress and immunosuppression can modulate inflammatory responses, potentially accelerating disease progression [49]. Our investigation considers how these factors interact with systemic inflammation to impact symptom perception and the timeliness of medical presentation in patients with NMSC and CRC.

Recognizing the practical needs in clinical settings, our study aims to develop a risk assessment tool based on the identified patient profiles. By integrating molecular profiling with clinical and psychosocial data, we endeavor to create a questionnaire that assists clinicians in stratifying patients according to risk levels. This tool is intended to facilitate the early identification of high-risk individuals, enabling personalized interventions and potentially improving clinical outcomes.

The primary objective of this study is to employ a comprehensive approach that combines traditional statistical methods with neural network analyses to profile patients with NMSC and CRC based on inflammatory markers, sociodemographic variables, and psychological factors. Through this comparative analysis, we aim to identify common patterns and distinct differences in how these factors influence cancer progression and patient behavior regarding healthcare engagement. This approach has the potential to enhance intervention strategies, promote early detection, and support the implementation of personalized treatments, ultimately improving the quality of life for patients.

### 1.4. Major Contributions

This study offers several significant contributions to the field of oncology and personalized medicine:

1. Integrated profiling approach: By combining traditional statistical methods with machine learning techniques, this research develops a comprehensive, multi-dimensional profiling model for patients with NMSC and CRC. The model examines the interplay of clinical, sociodemographic, and psychological factors on cancer progression, providing a holistic understanding of patient profiles.
2. Comparative analysis of cancer types: This study conducts a comparative analysis between NMSC and CRC, two cancers with distinct clinical manifestations. This comparison identifies both overlapping and unique patterns in how biological and psychosocial factors influence disease outcomes across different cancer types, thereby enhancing the understanding of cross-cancer influences and mechanisms.
3. Development of a practical risk assessment tool: A key innovation of this study is the creation of a cancer risk assessment questionnaire. This tool enables clinicians to classify patients into risk categories (low, moderate, high) based on identified profiles, facilitating early identification and personalized intervention strategies. The simplicity and comprehensiveness of the questionnaire ensure its practicality and applicability in diverse clinical settings.
4. Clinical relevance and personalized interventions: The findings highlight potential pathways for personalized interventions. Insights gained from patient profiling can guide the selection of targeted and effective treatment strategies, improving clinical outcomes. This study underscores the importance of addressing both inflammatory and psychosocial factors in the management of patients with cancer.
5. Framework for broader oncological applications: The robust profiling model developed through the comparison of NMSC and CRC can be adapted and applied to other cancer types. This versatility supports the advancement of personalized medicine across a wide range of malignancies, promoting early detection and tailored therapeutic approaches.

## 2. Materials and Methods

### 2.1. Study Design

**Figure 1:**
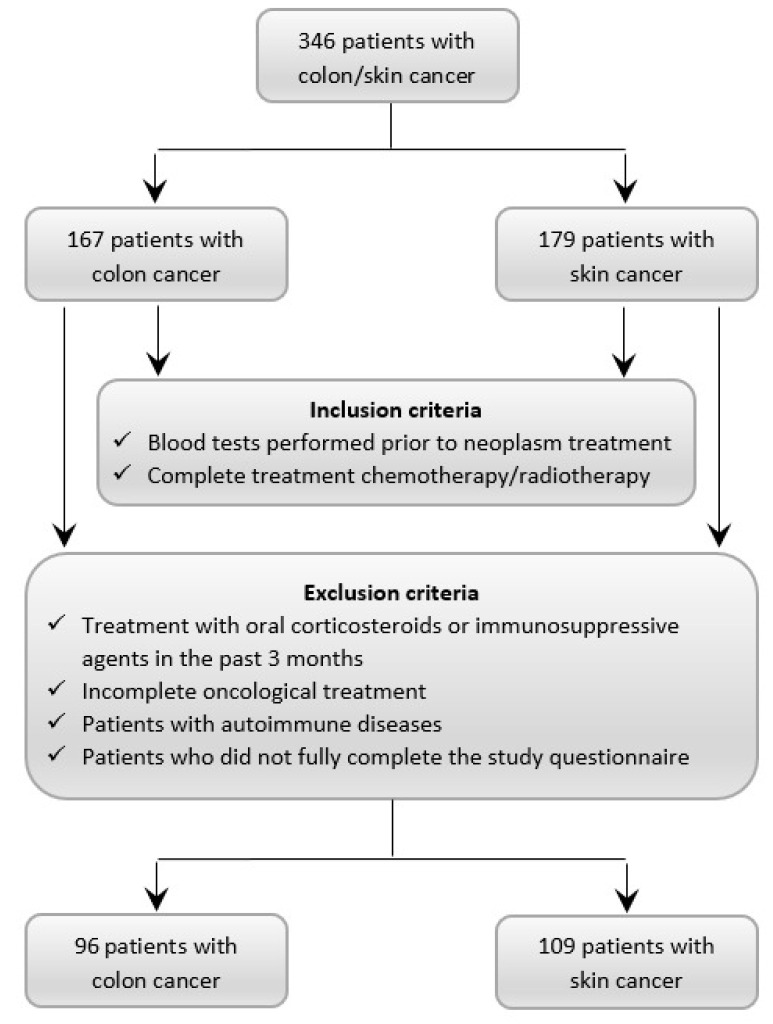
Study Flowchart.

This study employed a comparative retrospective methodology conducted over a three-year period, from 15 September 2020 to 14 August 2023. The research included consecutively admitted patients diagnosed with cutaneous carcinoma at the Plastic Surgery Clinic of Emergency County Hospital No. 1 in Craiova and colorectal neoplasm at the Surgery Clinic of Dr. Ștefan Odobleja Emergency Military Hospital. The initial study cohort comprised 167 patients with colorectal cancer, including cases across all segments of the colon (cecum, ascending colon, transverse colon, descending colon, sigmoid colon, and rectum), and 179 patients with cutaneous cancer (Figure 1).

### 2.2. Inclusion Criteria

To be included in this study, patients needed to have diagnosed with colorectal neoplasm or cutaneous carcinoma within the period between 15 September 2020 and 14 August 2023. It was essential that these patients had a complete blood count performed prior to the initiation of any treatment and had completed their therapeutic regimens, including chemotherapy, radiotherapy, or combinations thereof.

### 2.3. Exclusion Criteria

Patients were excluded from this study if they were treated with oral corticosteroids or immunosuppressive agents in the past three months, had incomplete oncological regimens, or were diagnosed with autoimmune diseases.

Although this study employed a retrospective methodology, a comprehensive anamnesis was conducted in the two clinics, involving extensive questioning. This thorough approach ensures that the discussion with the patient remains a critical element in achieving the most accurate diagnosis possible. The anamnesis was designed to capture a wide range of sociodemographic, psychological, and clinical factors, which are essential for the nuanced profiling and management of patients.

### 2.4. Data Collection

For each patient, relevant demographic and clinical data were collected, including the following:

Demographic Information: gender, age, and place of residence.

Clinical and Socioeconomic Characteristics: skin color, smoking status, tumor stage, sun exposure, living conditions, marital status, family context, stress level, and frequency of anxiety and panic episodes. In cases where patients were unable to provide this information due to medical or other reasons, data were collected from close relatives who had familial or legal standing as caregivers.

Blood Count Results: obtained within the first 24 h of hospitalization, including parameters such as white blood cell count, hematocrit, lymphocyte count, monocyte count, neutrophil count, platelet count, red cell distribution width, mean corpuscular volume, and total serum proteins.

Patient Outcomes: discharge status was also documented.

These data were used to create a comprehensive profile of the inflammatory, environmental, sociodemographic, and psychological factors in non-melanoma skin cancer and colorectal cancer. To ensure data accuracy and integrity in this retrospective study, data entries were cross-checked by two independent researchers, and regular consistency checks were conducted to identify and correct discrepancies. Data collection adhered to standardized protocols and validated questionnaires. Patients for whom complete data could not be obtained, either from themselves or their relatives, were excluded from this study to maintain consistency and reliability.

This study was approved by the Ethics and Scientific Deontology Committee of the University of Medicine and Pharmacy Craiova (no. 115/15 June 2022), ensuring adherence to the highest ethical and scientific standards. Informed consent was obtained from all participants or their legal guardians, in compliance with ethical guidelines for retrospective studies.

### 2.5. Statistical Analysis

Statistical analysis in this study was meticulously conducted using SPSS software, version 26.0. (IBM Corporation, Armonk, NY, USA) Initially, detailed descriptive statistics were established for the entire study population, providing a thorough framework for subsequent evaluations. Normally distributed values were expressed as mean ± standard deviation (±SD), while categorical variables were presented as percentages. In the context of assessing the impact of inflammatory indices on complications or mortality, the independent t-test was employed as the primary statistical tool.

For variables that did not meet the assumption of normality, the Mann–Whitney U test was used to compare medians between groups. This non-parametric test is particularly suited for small sample sizes or skewed data distributions, as it does not rely on the assumption of normality. Additionally, the Fisher Exact test was employed for categorical variables with low expected frequencies, as it provides an exact probability for observing the data, ensuring accurate statistical inference even in cases with small sample sizes. These statistical methods were selected to ensure accurate and dependable comparisons, accounting for the unique characteristics of the data.

Although traditional analysis highlighted certain significant differences between cancer types and stages, the high variability and lack of uniformity in the results suggested the need for a more advanced approach to identify clearer patterns and patient subgroups. Consequently, we resorted to machine learning techniques, particularly clustering, to uncover homogeneous patient groups based on multiple variable sets, offering a new perspective on our data.

To improve the model’s performance and avoid the risk of overfitting, we applied dimensionality reduction techniques, particularly Principal Component Analysis (PCA). This method transformed the original variables into a set of uncorrelated principal components, effectively reducing the total number of variables. We selected only those components that explained a significant proportion of the variance (for example, a cumulative variance threshold of 95%), ensuring that only the most statistically relevant variables were retained. This approach simplified the input into the neural network and reduced the risk of model complexity. Patient profiling was performed using Python 3.11 and machine learning libraries such as scikit-learn versiosn 1.5.2.

#### 2.5.1. Preprocessing of Features

Data preprocessing involved several steps to ensure consistency and accuracy. Continuous variables were standardized using StandardScaler, while categorical variables were encoded using OneHotEncoder to handle non-numeric data. Additionally, data-cleaning procedures were applied to handle missing values and outliers, ensuring a clean and reliable dataset before feeding it into the neural network. This preprocessing step was crucial for obtaining clusters of patients with similar characteristics, leading to a deeper understanding of patient profiles and how different variables—biological, psychological, and socio-demographic—interact in the context of cancer.

#### 2.5.2. Type of Neural Network

**Figure 2:**
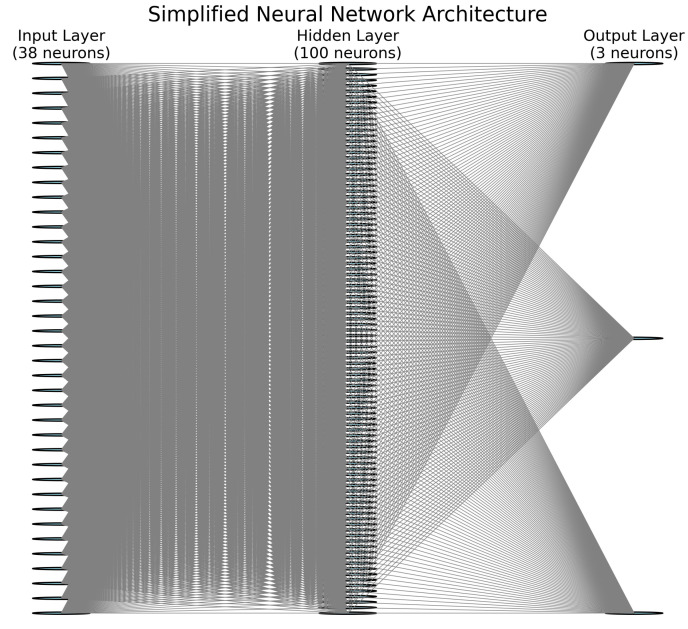
Feedforward neural network structure.

The neural network model, designed to classify patient profiles across both non-melanoma skin cancer and colorectal cancer, was built as a Feedforward Neural Network (Multilayer Perceptron—MLP). The architecture consists of an input layer with 38 neurons corresponding to preprocessed patient features, a single hidden layer with 100 neurons, and an output layer with 3 neurons representing the distinct patient profiles. The hidden layer uses the ReLU activation function, while the output layer employs the Softmax function for multiclass classification (Figure 2).

This architecture allowed us to capture the complex, non-linear relationships between biological, psychological, and socio-demographic variables, ensuring accurate classification and profiling of patient subgroups.

#### 2.5.3. Validation of the Neural Network Model

To validate the neural network model and evaluate its ability to generalize well on unseen data, we applied k-fold cross-validation. We used a 5-fold cross-validation, which divided the data into five equal subsets. Each subset was alternately used as a test set, while the remaining four subsets were used to train the model. This process allowed us to prevent overfitting and assess the reliability of the predictions.

For evaluating the model’s performance, we used the following metrics:

- Accuracy, to measure the percentage of correct predictions.
- Recall, to evaluate the model’s ability to identify high-risk patients.
- Precision, to measure the accuracy of positive predictions.
- F1-score, a performance metric that combines recall and precision.

These metrics were aligned with the objectives of our study, focusing on correctly identifying patients with biological and psychological characteristics relevant to cancer progression. The model’s performance on the test data demonstrated good generalization capabilities, ensuring that the model learned meaningful relationships between variables without excessively fitting to the training data.

#### 2.5.4. Capturing Variable Interactions in the Neural Network Model

In addition to validating the model’s generalization, we focused on how well the neural network could capture complex interactions between different types of variables.

The interaction between biological factors (such as inflammation), psychological factors (like stress and anxiety), and socio-demographic factors (including family support and living conditions) was central to this study. The neural network model was designed to capture these interactions, both synergistic and antagonistic, between these diverse types of variables.

- Capturing Interactions: The neural network architecture allows for the detection of non-linear relationships and interdependencies between variables. For example, patients with both high levels of stress and elevated inflammation markers tend to exhibit distinct patterns in disease progression. These complex interactions were captured by training the neural network on both continuous and categorical variables, allowing the model to learn the joint effects of these factors on cancer outcomes.
- Synergistic and Antagonistic Effects: The model was able to capture synergistic effects between psychological stress and inflammation, where the presence of both factors amplified the severity of disease progression. Conversely, it could also identify antagonistic effects, such as the protective role of a stable family environment, which mitigated the negative impacts of high stress and poor living conditions.

#### 2.5.5. Advanced Clustering for Patient Profiles

To categorize patients into homogeneous groups based on their inflammatory, sociodemographic, and psychological profiles, we employed an advanced clustering methodology. We selected the K-means algorithm due to its efficiency in partitioning data based on feature similarity. The optimal number of clusters was determined using the Elbow Method, which ensured a balance between intra-cluster similarity and inter-cluster dissimilarity.

Euclidean distance was chosen as the primary metric to measure similarity between data points, facilitating effective cluster formation. The consistency of the clusters was evaluated using silhouette analysis, which compares the cohesion within clusters to the separation between them. High silhouette scores indicated that the clusters were well-defined and distinct.

To validate the identified patterns, we integrated the clustering results with outputs from neural networks. This dual approach enhanced the reliability of our patient profiling by ensuring that both unsupervised and supervised learning methods corroborated the findings. After the neural network captured the complex interactions among biological, psychological, and sociodemographic variables, we applied the K-means clustering technique to group patients with similar profiles. In our study, we selected three clusters (k = 3) to identify distinct patient profiles, initializing the algorithm with ten centroid starting points (n_init = 10) and setting a random state of 42 for reproducibility.

This clustering analysis successfully classified patients into distinct subgroups, each representing unique combinations of the measured variables. This classification provided a deeper understanding of how various factors interact in the context of cancer, enabling the identification of patient subgroups with similar patterns of disease progression.

## 3. Results

This study initially included 346 patients, of whom 179 were diagnosed with skin cancer and 167 with colon cancer. After applying the inclusion and exclusion criteria, the final cohort consisted of 205 patients, divided into two subgroups: 109 patients diagnosed with skin cancer (53.17% of the final cohort) and 96 patients diagnosed with colon cancer (46.83% of the final cohort). The ages of the patients ranged from 23 to 91 years. The mean age of the patients with skin cancer was slightly lower compared to that of the patients with colon cancer, with values of 64.71 ± 15.20 years and 66.18 ± 10.37 years, respectively. However, the differences between the two groups were not statistically significant (Table 1). Both groups were similar in terms of gender, place of residence, smoking status, and skin type (*p* > 0.05) (Table 2).

A significant proportion of the patients in the skin cancer group reported high sun exposure (89 patients, or 81.65% of the total), while in the colon cancer group, the distribution was more balanced.

Among the patients with skin cancer, tumor stage T1 was the most frequently diagnosed (41 patients, or 37.61%), followed by stages T2 (34 patients, 31.19%), T3 (25 patients, 22.92%), and T4 (9 patients, 8.26%). In contrast, among the patients with colon cancer, stage T3 was the most common (47 patients, 48.96%), followed by stages T2 (38.54%), T4 (10.42%), and T1 (2.08%). The observed differences in tumor stages between the two groups were statistically significant (*p* < 0.0005).

Due to the significant differences observed in tumor stages between patients with skin cancer and colon cancer, further analysis focused on sociodemographic and biological parameters according to tumor stage, as these differences may offer valuable insights into disease understanding and management.

To investigate differences between the two subgroups (skin cancer and colon cancer) regarding clinical parameters, the Mann–Whitney U test was used. Although these cancers affect different tissues, both are influenced by systemic inflammation and immune response, which are key factors in disease progression. By comparing clinical parameters between these two cancer types, we aim to identify both common patterns and distinct differences in how these factors affect cancer development and progression. Although no significant differences were observed in the mean leukocyte count between groups, important variations were identified in other aspects. Neutrophil levels were significantly higher in patients with colon cancer (*p* = 0.003), while lymphocytes were more abundant in patients with skin cancer (*p* < 0.001), suggesting differences in immune response. In terms of coagulation and metabolic profiles, patients with skin cancer exhibited a higher mean platelet count (*p* = 0.001) and elevated protein levels (*p* < 0.001) compared to those with colon cancer. These variations may reflect particular physiological processes in the two types of cancer. Erythrocyte indicators, such as RDW and MCV, also showed significant differences, with RDW slightly higher in skin cancer (*p* = 0.044) and MCV showing significant variations between groups (*p* = 0.002).

Cellular ratios, such as NLR (neutrophil-to-lymphocyte ratio) and dNLR (derived neutrophil-to-lymphocyte ratio), showed significant differences (*p* = 0.028 for NLR and *p* < 0.001 for dNLR), indicating variations in immune response. Although PLR (platelet-to-lymphocyte ratio) did not show a significant difference (*p* = 0.202), other indicators like IIC (cumulative inflammatory index) and MCVL (MCV-to-lymphocyte ratio) showed significant variations (*p* = 0.001 for IIC and *p* = 0.089 for MCVL) (Table 3).

### 3.1. Comparative Analysis of Sociodemographic and Psychological Factors in Skin Cancer by Tumor Stage

In the analysis of patients with skin cancer, there was a predominance of men in the advanced stages, though without statistical significance. Patients from rural areas tended to be diagnosed at advanced stages, but this, too, lacked statistical significance. Living conditions and stress levels were significantly associated with advanced stages of the disease (*p* < 0.01), highlighting a connection between these factors and tumor progression.

### 3.2. Comparative Analysis of Sociodemographic and Psychological Factors in Colon Cancer by Tumor Stage

In colon cancer, gender distribution was balanced, with no statistically significant differences between stages. The mean age of patients was significantly higher in stage II (*p* < 0.01). A stable family environment and high-stress levels were significantly associated with advanced stages of the disease (*p* < 0.05), suggesting important influences of these factors on tumor progression.

### 3.3. Comparative Analysis of Sociodemographic and Psychological Factors in Both Cancer Types by Tumor Stage

This study highlighted significant differences in sociodemographic and psychological factors according to tumor stages for both skin and colon cancer. Although non-melanoma skin cancer and colorectal cancer affect different tissues, these cancers share common biological mechanisms, particularly systemic inflammation and immune response, which play a key role in disease progression. Additionally, socio-demographic factors such as living conditions, stress levels, and family environment similarly influence cancer outcomes across both types. By comparing these cancers, we aim to identify common patterns and differences in how these factors interact with tumor stage and progression. Living conditions and family environment were significantly associated with disease progression in both cancer types (*p* < 0.01), while stress levels and panic reactions had differentiated influences between these cancers (*p* < 0.05). These findings underscore the complex role of psychosocial factors in tumor evolution (Table 4).

### 3.4. Comparative Analysis of Biological Parameters in Skin Cancer by Tumor Stage

The analysis of biological parameters in skin cancer showed significant variations as the disease progressed. Leukocytes and neutrophils increased significantly in stage IV (*p* < 0.05), and platelet and protein levels also showed elevated values (*p* < 0.05). NLR and dNLR ratios displayed variations, suggesting their involvement in tumor progression (Table 5).

### 3.5. Comparative Analysis of Biological Parameters in Colon Cancer by Tumor Stage

For colon cancer, leukocytes and neutrophils increased significantly in stage IV (*p* < 0.05), while lymphocytes progressively decreased (*p* < 0.001). RDW and MCV showed significant variations (*p* < 0.001), and dNLR and PLR ratios revealed notable differences, suggesting possible implications in tumor progression.

### 3.6. Comparative Analysis of Biological Parameters in Both Cancer Types by Tumor Stage

The comparative analysis revealed significant differences between skin and colon cancer regarding leukocytes, neutrophils, and lymphocytes (*p* < 0.05). Inflammatory markers such as NLR and dNLR showed notable variations between cancer types, indicating possible immunological distinctions. Additionally, PLR and MCVL displayed significant differences, suggesting particularities in disease progression between the two cancer types.

Although the comparative analysis of biological parameters by tumor stage provided an overview of the differences between the two cancer types, this approach offered primarily descriptive data and highlighted only limited statistical significance. These findings, while informative, do not fully capture the underlying complexity of the interactions between the diverse clinical, biological, and psychosocial factors involved. The high variability and absence of clear patterns suggest that analyzing these parameters separately from the broader context of influencing factors limits their clinical utility. Consequently, we transitioned to a more advanced machine learning approach, employing neural networks to profile patients more comprehensively. This method allows for the integration of multiple variables, which interact in complex ways, to better understand patient subgroups and their unique cancer progression dynamics.

### 3.7. Patient Profiles in Non-Melanoma Skin Cancer

#### 3.7.1. Stable Profile in Patients with Non-Melanoma Skin Cancer (Profile 0)

Patients within the Stable Profile for non-melanoma skin cancer have a mean age of 64.71 years (SD = 11.85 years), indicating a predominantly middle-aged to elderly population. The average white blood cell (WBC) count is 6.57 (SD = 1.82), reflecting moderate variation in the inflammatory response. Neutrophils have an average value of 3.99, and lymphocytes average 1.71, suggesting a balanced immune status with no signs of severe infection or dysfunction. The platelet count averages 273.66 (SD = 115.76), highlighting variability in blood coagulation, possibly influenced by treatment or the inflammatory state of the disease. Inflammatory markers such as NLR (neutrophil-to-lymphocyte ratio) and PLR (platelet-to-lymphocyte ratio) have average values of 2.53 (SD = 0.58) and 165.53 (SD = 75.30), respectively, indicating moderate inflammation. The cumulative inflammatory index (CII) stands at 2.78, suggesting slightly above-average inflammatory activity (Table 6).

From a categorical perspective, the majority of patients are in the early stages of the disease, with 50.85% in stage 1 and 37.29% in stage 2, indicating a relatively favorable prognosis. A stable family environment is reported by 93.22% of patients, indicating strong social support. Regarding living conditions, 87.93% of patients report adequate or better living standards, which may influence access to healthcare and quality of life. Maritally, 81.36% of patients are married or in a relationship, providing consistent psychosocial support. However, 54.24% of patients experience moderate to high levels of stress, and 45.76% report frequent episodes of anxiety and panic, reflecting the psychological impact of a cancer diagnosis (Table 7).

#### 3.7.2. High-Risk Profile in Patients with Non-Melanoma Skin Cancer (Profile 1)

**Figure 3:**
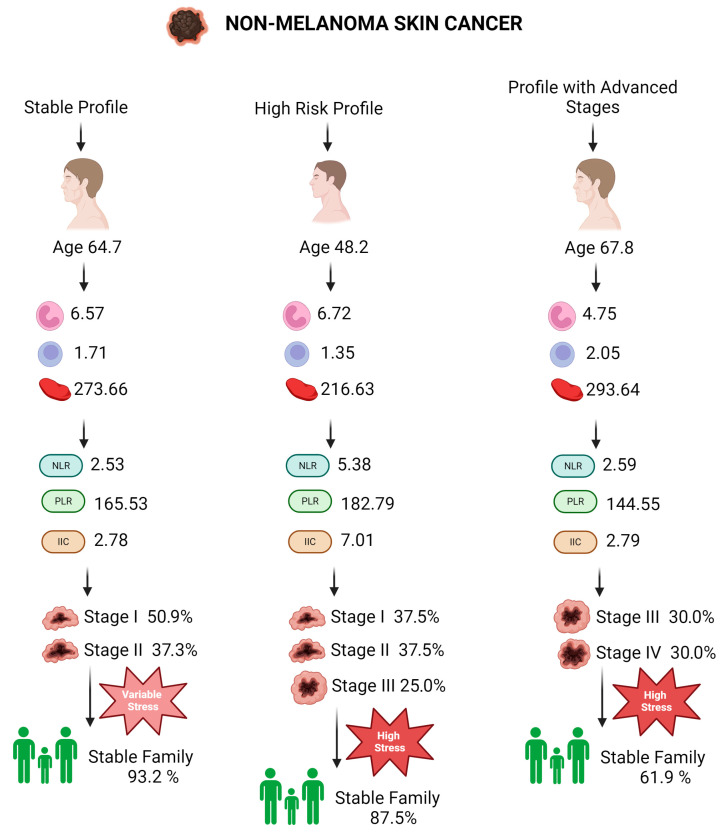
Profiles of patients with non-melanoma skin cancer: Comparison of Stable Profile, High-Risk Profile, and Advanced-Stage Profile—created with BioRender.com.

Patients in the High-Risk Profile have a mean age of 48.25 years (SD = 16.80 years), indicating a wide age distribution within this group. The blood marker profile shows an average WBC count of 8.84 (SD = 1.69), indicating potential acute inflammatory states. Neutrophils average 6.72, while lymphocytes average 1.35, suggesting a marked inflammatory response and a potentially suppressed immune reaction. The platelet count averages 216.63 (SD = 27.78), reflecting relative stability but at a reduced level. Inflammatory markers such as NLR, PLR, and dNLR (derived neutrophil-to-lymphocyte ratio) are significantly elevated, with average values of 5.38, 182.79, and 3.81, respectively, emphasizing an intense inflammatory response. The cumulative inflammatory index (CII) is very high, with an average of 7.01, reflecting marked systemic inflammation (Figure 3).

Categorically, this profile shows a relatively balanced distribution across tumor stages, with 37.5% of patients in stage 1, 37.5% in stage 2, and 25% in stage 3. Most patients, 87.5%, have a stable family environment, which may contribute to adequate social support in managing the disease. Regarding living conditions, 75% of patients report average or better living conditions, suggesting a sufficient material support base. Maritally, 50% of patients are married, and 37.5% are in a relationship, offering thorough social support. However, 75% of patients report high levels of stress, and 62.5% experience frequent anxiety and panic episodes, underscoring a significant psychological impact.

#### 3.7.3. Advanced-Stage Profile in Patients with Non-Melanoma Skin Cancer (Profile 2)

Patients in the Advanced-Stage Profile have a mean age of 67.83 years (SD = 17.29 years), indicating a broad age range from midlife to elderly. The average WBC count is 8.01, with neutrophils averaging 4.75, suggesting a moderate inflammatory response, characteristic of chronic inflammatory diseases or latent infections. Lymphocytes average 2.05, indicating an active immune response. The platelet count averages 293.64 (SD = 122.71), reflecting variability in response to inflammation or side effects of therapies. Inflammatory markers such as NLR, PLR, and dNLR have average values of 2.59, 144.55, and 1.53, respectively, indicating a consistent and active inflammatory response. The cumulative inflammatory index (CII) averages 2.79, suggesting moderate inflammation.

Regarding categorical variables, nearly 60% of patients are in the advanced stages of the disease (stages 3 and 4), indicating a prevalence of more severe forms of the disease. The family environment is unclear for 38.10% of patients, which may complicate social support and disease management. Most patients (78.57%) live in adequate living conditions, which might reflect limited access to high-quality healthcare resources. Maritally, 50% of patients are single, indicating potentially reduced social support. High levels of stress (70.24%) and anxiety (61.90%) are common in this profile, with 54.76% of patients experiencing frequent panic episodes, suggesting a considerable psychological impact that needs to be addressed through appropriate psychosocial interventions.

### 3.8. Patient Profiles in Colon Cancer

#### 3.8.1. Stable Profile in Patients with Colon Cancer (Profile 0)

Patients with the Stable Profile for colon cancer have a mean age of 69.03 years (SD = 8.78 years), indicating a predominantly elderly population. The interquartile range (IQR) between 63 and 73 years suggests a concentrated age distribution. The average white blood cell (WBC) count is 6.55 (SD = 1.45), with neutrophils at 4.11 and lymphocytes at 1.74, indicating a well-balanced immune response. The platelet count averages 252.88 (SD = 123.00), reflecting variability in the inflammatory response. Total proteins and mean corpuscular volume (MCV) are within normal ranges, with averages of 6.47 and 90.16, respectively, indicating proper nutrition and liver function. Inflammatory markers NLR, PLR, and dNLR have average values of 2.77, 145.43, and 1.72, respectively, and the cumulative inflammatory index (CII) stands at 2.97, suggesting a moderate degree of chronic inflammation (Table 8).

Categorically, the majority of patients (93.94%) are in stage 2 of the disease, indicating a localized tumor and the potential for effective treatment. These patients, being in intermediate stages, have a higher likelihood of successful treatment due to the limited spread of the disease. A stable family environment, reported by 87.88% of patients, and good living conditions (81.82%) are factors that contribute to strong social support, essential for managing the disease. The majority of patients (84.85%) are married, which provides important emotional and logistical support, facilitating treatment adherence and improving quality of life. However, 72.73% of patients experience moderate to high levels of stress, and 66.67% report frequent episodes of anxiety and panic, highlighting a significant psychological impact that requires appropriate stress management and psychosocial support interventions (Table 9).

#### 3.8.2. High-Risk Profile in Patients with Colon Cancer (Profile 1)

**Figure 4:**
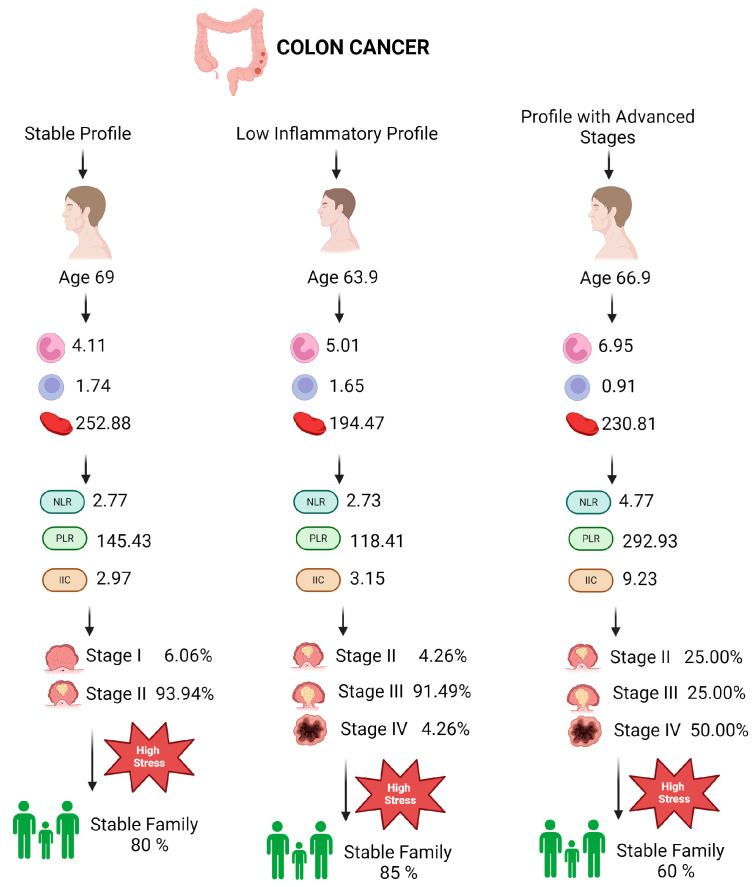
Profiles of patients with colon cancer: Comparison of Stable Profile, Low-Inflammation Profile, and Advanced-Stage Profile—created with BioRender.com.

Patients in the High-Risk Profile have a mean age of 63.91 years (SD = 10.63 years), reflecting a predominantly middle-aged to elderly population, with an IQR between 59.50 and 71.50 years. The average WBC count is 7.08 (SD = 1.24), with neutrophils at 5.01, suggesting heightened inflammatory activity. Lymphocytes average 1.65 (SD = 0.19), indicating a stable immune response. The platelet count averages 194.47 (SD = 73.31), and total proteins are lower, with an average of 4.66 (SD = 0.99), suggesting possible nutritional deficiencies. Inflammatory markers NLR, PLR, and dNLR have average values of 2.73, 118.41, and 1.87, respectively, while the CII presents a higher value of 3.15, indicating persistent inflammation (Figure 4).

Categorically, this profile is characterized by advanced disease stages, with 91.49% of patients in stage 3 of colon cancer. This indicates tumor progression, necessitating more aggressive treatment and continuous monitoring. In terms of family environment, 80.85% of patients report a stable family setting, which can provide consistent support during treatment. About 76.60% of patients have adequate or better living conditions, facilitating access to medical services and better recovery. Maritally, 80.85% of patients are married, offering essential emotional support needed to cope with the stress associated with treatment and the disease itself. However, a significant percentage of 80.85% of patients experience high levels of stress, and 76.60% report frequent episodes of anxiety and panic, highlighting a major psychological impact that requires psychological and social interventions to improve quality of life and treatment effectiveness.

#### 3.8.3. Advanced-Stage Profile in Patients with Colon Cancer (Profile 3)

Patients in the Advanced-Stage Profile have a mean age of 66.94 years (SD = 11.66 years), indicating a middle-aged to elderly population, with an IQR between 60.75 and 76.50 years. The average WBC count is 8.43 (SD = 0.94), and neutrophils average 6.95, indicating active acute or chronic inflammation. Lymphocytes, with an average of 0.91 (SD = 0.31), suggest severe immune suppression. The platelet count averages 230.81 (SD = 99.33), and total proteins are lower, with an average of 5.71 (SD = 1.23), indicating potential nutritional deficiencies. Inflammatory markers NLR, PLR, and dNLR are extremely elevated, with average values of 4.77, 292.93, and 5.48, respectively, suggesting an intense inflammatory response, while the CII presents an extremely high value of 9.23, reflecting severe inflammation.

Categorically, this profile is marked by very advanced disease stages, with 50% of patients in stage 4, indicating extensive metastasis and a reserved prognosis. A stable family environment is reported by 68.75% of patients, which can provide essential emotional support in managing advanced disease. However, 31.25% of patients reported an unstable or unclear family environment, which can complicate disease management and negatively affect quality of life. Living conditions are reported as adequate or better by 75% of patients, facilitating access to necessary resources for treatment and recovery. Maritally, 81.25% of patients are married, providing important social support, yet 81.25% of these patients report high levels of stress, and 75% experience frequent episodes of anxiety and panic, reflecting a severe psychological impact that requires specialized psychosocial interventions to improve quality of life and reduce the emotional burden.

#### 3.8.4. Evaluation of Model Performance

The neural network model, designed to classify patient profiles across both non-melanoma skin cancer and colorectal cancer, was evaluated based on several performance metrics, including accuracy, precision, recall, and F1-score. On the test set, the model achieved an overall accuracy of 98%, indicating that almost all cases across both cancer types were classified correctly. Precision and recall were similarly high, each at 98%, suggesting that the model was able to make accurate predictions and correctly identify the majority of real cases across profiles. The F1-score, a metric that combines precision and recall, was also 98%, confirming a balanced and thorough performance.

**Figure 5:**
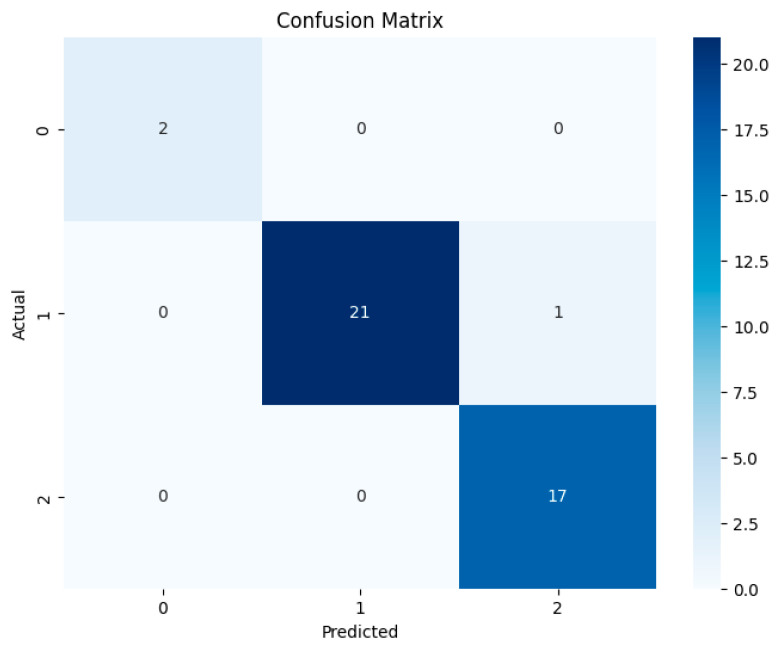
Confusion matrix of the neural network model predictions.

The confusion matrix demonstrates that the model correctly classified all cases for Profiles 0 and 2, while one instance in Profile 1 was misclassified as Profile 2, indicating a slight similarity between these two profiles (Figure 5).

**Figure 6:**
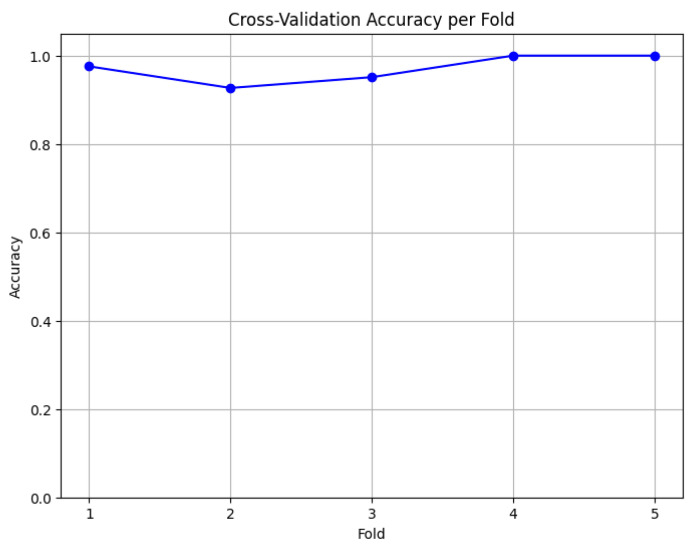
Cross-validation accuracy scores across 5 folds.

To assess the model’s generalization capability, 5-fold cross-validation was applied. The accuracy for each fold ranged between 92.68% and 100%, with the following values: 97.56% for Fold 1, 92.68% for Fold 2, 95.12% for Fold 3, and 100% for Folds 4 and 5. The average accuracy across the cross-validation was 97%, reflecting thorough performance and consistent generalization of the model across different data subsets (Figure 6).

**Figure 7:**
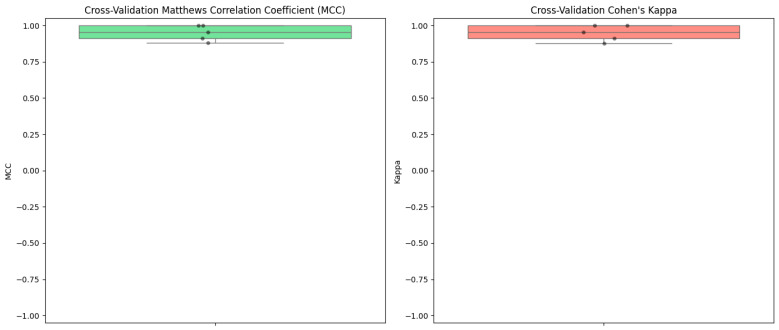
Matthews Correlation Coefficient (MCC) and Cohen’s Kappa scores across 5-fold cross-validation.

In addition to the primary performance metrics, the Matthews Correlation Coefficient (MCC) and Cohen’s Kappa were calculated to provide a more comprehensive evaluation of the model’s performance (Figure 7).

These metrics are particularly valuable as they account for the balance between classes and the possibility of agreement occurring by chance.

Across the 5-fold cross-validation, the MCC scores were as follows: 0.956, 0.879, 0.910, 1.000, and 1.000. This resulted in a mean MCC of 0.95 with a standard deviation of 0.05. Similarly, Cohen’s Kappa scores for each fold were 0.955, 0.877, 0.910, 1.000, and 1.000, yielding a mean Kappa of 0.95 and a standard deviation of 0.05. These high values for both MCC and Kappa indicate a strong correlation between the predicted and actual classifications, as well as a high level of agreement beyond what would be expected by chance.

The inclusion of MCC and Cohen’s Kappa reinforces the conclusion that the neural network model not only performs exceptionally well in terms of traditional metrics but also maintains robust and reliable classification performance across different data subsets. These additional metrics highlight the model’s ability to handle class imbalances and ensure that the predictions are both accurate and consistent, further validating its suitability for patient profile classification.

These results indicate that the neural network model is well-suited for patient profile classification and can efficiently handle data variations. The minor confusion between Profiles 1 and 2 suggests that the model could benefit from slight adjustments to improve the separability between these two profiles. However, the overall performance remains excellent, with a strong ability to generalize to unseen data.

## 4. Discussions

Although classical statistical analysis was initially employed to evaluate the relationships between inflammatory and psychological factors and cancer progression in this study, the results revealed significant limitations. Specifically, the statistical significance varied considerably across different cancer types and parameters studied, indicating that the complex interactions between these factors may not be adequately captured by traditional methods. This variability suggests that biological mechanisms and individual responses can differ significantly between patients, making it challenging to interpret the data clearly through univariate or bivariate analysis. To avoid reiterating conclusions already established in the literature and to provide a more nuanced perspective, we turned to neural networks to identify complex patient profiles and highlight subtle patterns that were not evident in the initial analysis.

While our analysis highlights the significant impact of stress, depression, and familial issues on the progression and management of advanced cancer stages, it is also important to recognize that the challenges posed by advanced disease can intensify these psychosocial factors. This bidirectional relationship underscores the complexity of cancer progression and the necessity for comprehensive patient support systems that address both medical and psychological needs. However, due to the retrospective and observational nature of our study, we cannot definitively establish causality between these variables.

### 4.1. Analysis of the Stable Patient Profile

#### 4.1.1. Stable Profile in Non-Melanoma Skin Cancer

Patients with non-melanoma skin cancer in this profile are characterized by a middle-aged demographic and low levels of inflammation (NLR: 2.53, PLR: 165.53). These low levels of inflammation suggest a relatively balanced immune system, which may be attributed to a healthy lifestyle and the absence of major risk factors. A stable family environment plays a crucial role in maintaining this balance, contributing to health awareness and the adoption of preventive behaviors.

Chronic inflammation is a significant factor in cancer progression. For instance, elevated levels of inflammatory markers like IL-1β and CXCL10 are associated with an increase in immunosuppressive cells, thereby promoting disease progression [50]. In contrast, a healthy lifestyle and family support can reduce systemic inflammation, leading to a better prognosis and improved response to treatments [51,52].

The diagnosis of non-melanoma skin cancer in this profile is often made at early stages (Stage I: 50.85%, Stage II: 37.29%), largely due to the visibility of skin lesions and a family environment that supports early symptom recognition. Public awareness campaigns play a critical role in educating the population on the importance of self-examination and symptom recognition, thereby facilitating early detection of skin cancer [53,54]. Studies highlight that regular self-examination and public education are essential for improving prognosis through the early detection of malignant lesions [55,56].

A stable family environment, present in 93.22% of patients, and low levels of stress are important factors contributing to improved prognosis. Family support can reduce perceived stress and promote healthy behaviors, positively impacting quality of life and health outcomes. Studies show that social and family support is a significant predictor of quality of life and stress reduction, contributing to better psychosocial functioning and reduced inflammation [57,58,58,59,60].

#### 4.1.2. Stable Profile in Colon Cancer

For colon cancer, patients in this profile are predominantly diagnosed at stage II (93.94%) and exhibit moderate levels of inflammation (NLR: 2.77, PLR: 145.43). These moderate levels of inflammation, along with the effectiveness of screening programs, contribute to the early detection of cancer. Studies emphasize that screening through fecal occult blood tests (FOBT) and colonoscopy can significantly reduce colorectal cancer mortality, underscoring the importance of these programs in cancer prevention and early detection [61,62,63,64].

A stable family environment and social support play a crucial role in facilitating access to the necessary resources for prevention and disease management. Individuals with a family history of colorectal cancer are more likely to undergo screening procedures, reflecting increased risk awareness and active involvement in preventive measures [65]. Additionally, family support is fundamental for treatment adherence and improving patients’ quality of life [66]. Educating families about care behaviors can significantly enhance adherence to treatment regimens and the quality of care provided [67].

Additionally, it is essential to acknowledge that the progression to advanced stages of colon cancer can intensify stress and depression among patients. The increasing physical demands of the disease and concerns about treatment efficacy and prognosis may further exacerbate existing psychosocial challenges.

### 4.2. Analysis of the High Inflammatory Risk Profile

#### 4.2.1. Inflammatory Risk in Non-Melanoma Skin Cancer

Patients in the High Inflammatory Risk Profile exhibit elevated levels of inflammation (NLR: 5.38, PLR: 182.79), which may result from genetic or environmental factors, such as UV radiation exposure. Increased inflammation is associated with a heightened risk of developing and progressing non-melanoma skin cancer. UV radiation, responsible for approximately 90% of non-melanoma skin cancer cases, contributes to DNA damage and the activation of oncogenes, promoting the survival and uncontrolled proliferation of keratinocytes [68].

Research underscores that inflammation plays a crucial role in skin cancer progression, being involved in complex pathways of apoptosis, DNA repair, and immune signaling [69]. Genetic factors, such as variations in DNA repair genes, and chronic UV exposure are correlated with an increased risk of non-melanoma skin cancer [70]. Occupational UV exposure, particularly in individuals with fair skin, significantly raises the risk of squamous cell carcinoma, highlighting the need for adequate sun protection measures [71].

Although patients in the High Inflammatory Risk Profile exhibit high levels of inflammation, they are often diagnosed in the early stages (I and II). The visibility of symptoms and a certain level of awareness contribute to early medical consultations. However, chronic inflammation can affect both early detection and disease prognosis, negatively influencing immune response and accelerating tumor growth. Chronic inflammation is associated with elevated COX-2 and CRP levels in tumor tissues, indicating a significant role of inflammation in disease progression [72]. Additionally, chronic inflammation can lead to immune suppression, allowing tumors to evade immune destruction and compromising the effectiveness of treatments [73].

In 87.5% of cases, patients in this profile benefit from a stable family environment, but high-stress levels can exacerbate inflammation and negatively impact disease progression. Chronic inflammation combined with stress can accelerate cancer progression and reduce treatment efficacy. Studies show that psychological stress can amplify inflammatory responses through neuroendocrine factors such as catecholamines, which stimulate the production of pro-inflammatory cytokines [74]. Moreover, chronic stress and inflammation contribute to the accumulation of myeloid-derived suppressor cells (MDSCs), which block anti-tumor immune responses, thus promoting cancer progression [51].

Additionally, the demands of managing advanced cancer can strain family relationships and living conditions, thereby increasing psychosocial stressors and diminishing the support available to patients.

#### 4.2.2. Inflammatory Risk in Colon Cancer

Patients with colon cancer in the High Inflammatory Risk Profile exhibit moderate to severe inflammation (NLR: 2.73, PLR: 118.41), an indicator of disease progression and the risk of additional complications, including metastasis. Chronic inflammation is closely linked to advanced stages of colorectal cancer and metastasis, being associated with elevated inflammatory markers such as C-reactive protein (CRP) and IL-6 [75,76]. These inflammatory markers promote epigenetic changes and angiogenesis, facilitating the spread of tumor cells to other parts of the body [77]. Additionally, chronic inflammation, exacerbated by imbalances in gut microbiota, can contribute to the initiation and progression of colorectal cancer by influencing local inflammation and accelerating the oncogenic transformation of colonic epithelial cells [78].

Patients with colon cancer in this profile are often in stage III, where inflammation and a compromised immune response can accelerate disease progression. Intense local inflammation is associated with an anti-tumor immune response, positively influencing patient survival [79]. However, systemic inflammation, measured by biomarkers such as CRP and IL-6, is correlated with reduced survival and an increased likelihood of recurrence and death [80]. The interaction between local and systemic inflammation may provide a better prediction of survival using inflammation-based prognostic scores such as the Glasgow Prognostic Score (mGPS) [79].

An unstable family environment and high levels of stress can exacerbate inflammation and negatively influence treatment decisions and care-seeking behaviors. Chronic stress and anxiety can reduce quality of life and treatment adherence, worsening disease symptoms and complications [81,82]. Studies show that patients with strong family support are more actively involved in decision-making processes, which can improve treatment satisfaction and outcomes [83]. Stress can complicate treatment decision-making, leading patients to avoid discussions about treatment options and leaving decisions to doctors or family members [84].

Moreover, the complexities of managing advanced colon cancer can place additional strain on family dynamics and living environments, heightening psychosocial stressors and reducing the support network available to patients.

In conclusion, the High Inflammatory Risk Profile, characterized by elevated inflammatory risk, highlights the importance of managing inflammation and stress in the treatment of non-melanoma skin cancer and colon cancer. Chronic inflammation and stress not only exacerbate disease progression but can also influence treatment decisions and care-seeking behaviors. Psychosocial interventions and appropriate family support are essential for improving prognosis and quality of life in patients.

### 4.3. Analysis of the Advanced-Stage Patient Profile

#### 4.3.1. Advanced Non-Melanoma Skin Cancer Analysis

Patients in the Advanced-Stage Profile are often diagnosed in the later stages (III and IV) of non-melanoma skin cancer. This late diagnosis can be attributed to an unclear family environment and high-stress levels, which may delay medical consultations. The moderate inflammation observed in these patients may accelerate disease progression, suggesting an inefficient immune response.

Studies indicate that psychosocial barriers, such as inadequate education and low social cohesion, contribute to late diagnosis. In Colombia, these barriers were found to influence late diagnosis in 32.5% of cases, highlighting the need for equitable access to medical care [85]. Additionally, stress and anxiety can lead to avoidance of medical care, and patients with high-stress levels are more likely to ignore symptoms or delay seeking treatment [86,87]. Educational and psychosocial interventions can help overcome these barriers, reducing delays in diagnosis and improving prognosis [88].

Moderate inflammation in non-melanoma skin cancer may reflect an inefficient immune response, contributing to rapid disease progression. Chronic inflammation plays a crucial role in cancer initiation and progression, mediated by pathways such as NF-κB and STAT3. Inflammatory cytokines, such as TNF-α and IL-6, contribute to cell proliferation and metastasis [89]. Chronic inflammation is also associated with immunosuppression, facilitating the accumulation of myeloid-derived suppressor cells (MDSCs) and activation of the NALP3 inflammatory pathway, leading to decreased antitumor reactivity [90]. Increased COX-2 expression in non-melanoma skin cancer is linked to the production of prostaglandins, which promote tumor growth and metastasis [91].

An unclear family environment and high-stress levels (69.05%) can exacerbate symptom perception and care-seeking behaviors. The lack of clear family support may delay medical consultations and lead to diagnosis in advanced stages. Studies show that patients with adequate family support are more likely to engage in preventive behaviors and seek timely medical care, while chronic stress can reduce treatment adherence and worsen symptom perception [92,93,94].

Moreover, advanced stages of cancer may challenge family support systems, as the emotional and financial burdens of the disease can lead to increased familial stress and reduced capacity to provide adequate support to the patient.

#### 4.3.2. Advanced Colon Cancer Analysis

Patients with colon cancer in this profile exhibit severe inflammation (NLR: 4.77, PLR: 292.93, CII: 9.23), which may reflect a compromised immune response and preexisting chronic inflammation. Chronic inflammation is a key factor in the progression and metastasis of colon cancer, contributing to the creation of a tumor microenvironment conducive to the development of malignant cells [76]. Inflammatory mediators such as TNF-α and IL-6 promote cellular mutations and malignant transformation, facilitating metastasis [95].

Elevated inflammatory markers, such as CRP and pro-inflammatory cytokines, are indicators of advanced stages of colon cancer and are associated with a poor prognosis. Chronic inflammation not only supports tumor initiation but also increases angiogenesis and facilitates metastasis [75]. In the tumor microenvironment, inflammatory immune cells, such as tumor-associated macrophages (TAMs) and regulatory T cells (Tregs), can contribute to the suppression of anti-tumor immune responses, thereby promoting cancer progression [96].

Patients in this profile are often diagnosed in stages III and IV, influenced by an unstable family environment and high-stress levels, which may delay medical consultations and worsen prognosis. Studies show that patients with strong family support are more likely to make informed and active decisions regarding treatment, which improves treatment outcomes [97]. Social support is correlated with improved survival, and patients with adequate family support exhibit better treatment adherence and improved quality of life [98].

An unclear family environment and high-stress levels can lead to reduced symptom perception and delays in seeking treatment. Vagal tone, influenced by chronic stress, can mask symptoms or alter their perception, contributing to the late diagnosis of colon cancer [81,99]. Adequate psychosocial support and interventions to reduce stress are essential to improving patient prognosis and reducing late diagnosis [100].

Furthermore, it is important to recognize that the challenges and uncertainties associated with advanced cancer stages can, in turn, exacerbate stress and depression levels among patients. The physical burden of the disease, coupled with concerns about treatment outcomes and quality of life, may intensify existing psychological stressors.

In conclusion, the Advanced-Stage Profile highlights the complexity of managing non-melanoma skin cancer and colon cancer in advanced stages, within the context of an unclear family environment and high stress levels. Severe inflammation and reduced symptom perception contribute to rapid disease progression and a poor prognosis. Psychosocial support and educational interventions are essential for improving access to care, reducing delays in diagnosis, and enhancing the quality of life for patients.

### 4.4. Potential Influences of Sociodemographic and Psychological Factors on Symptom Perception and Medical Consultation in Patients with Non-Melanoma Skin Cancer

In this study, neural networks were employed to perform detailed patient profiling, analyzing how sociodemographic and psychological factors influence symptom perception and medical consultation behaviors among patients with non-melanoma skin cancer. This artificial intelligence approach enabled the identification of complex patterns and subtle interactions between variables, providing a deeper understanding than traditional statistical methods alone.

#### 4.4.1. Early Diagnosis Facilitated by Lesion Visibility

In non-melanoma skin cancer, early diagnosis is largely facilitated by the visibility of skin lesions. These lesions are often apparent and easily noticed by patients or their close contacts, leading to prompt medical consultation. The clear visibility of these lesions is a crucial factor in early detection, allowing for rapid medical intervention and preventing disease progression. Neural networks helped quantify and identify correlations between lesion visibility and diagnosis times, highlighting the importance of this aspect in our analysis [98].

#### 4.4.2. Impact of Skin Lesion Visibility

The visibility of skin lesions plays a pivotal role in the early detection of non-melanoma skin cancer. Patients or those around them can easily observe these changes in the skin, prompting quick visits to the doctor. This early observation is critical because lesions detected at early stages have a much higher survival rate compared to those detected at advanced stages. Studies show that visible skin lesions detected by patients are often in a more advanced stage than those observed by doctors, underscoring the importance of regular self-examination and public education [101].

Public awareness campaigns, such as those conducted by the British Association of Dermatology, have had a significant impact on educating the public about the warning signs of skin cancer, including non-melanoma lesions. These campaigns promote self-examination and regular doctor visits, thus contributing to early detection and reducing the incidence of advanced-stage cancer [53]. Educational initiatives like the Euromelanoma campaigns have proven highly effective in increasing early diagnosis rates of non-melanoma skin cancers, highlighting the importance of monitoring skin changes and consulting a dermatologist when in doubt [102].

#### 4.4.3. Systemic Inflammation and Early Detection

In the early stages of non-melanoma skin cancer, systemic inflammation is usually low. Inflammatory markers, such as NLR (neutrophil-to-lymphocyte ratio) and PLR (platelet-to-lymphocyte ratio), are at low levels, indicating a minimal inflammatory response to the initial tumor. This may facilitate the early recognition of symptoms by patients, without being masked by a generalized inflammatory response.

Research suggests that low levels of inflammatory markers like NLR and PLR can contribute to a clear perception of symptoms, enabling patients to notice subtle changes in their skin and seek treatment at early stages [103]. Minimal inflammation allows for easier detection of symptoms without interference from systemic inflammation, which can improve prognosis through early medical consultation [104]. Public awareness campaigns and patient education about the early signs of skin cancer can encourage self-examination and early medical consultation, thereby improving early detection rates [102].

#### 4.4.4. Reduced Symptom Severity Perception in Advanced Stages

In the advanced stages of non-melanoma skin cancer, patients may delay seeking medical help due to a reduced perception of symptom severity. This perception may be influenced by factors such as an unstable family environment and high levels of stress, which can diminish attention to symptoms or even lead to their disregard.

The family environment plays a crucial role in how patients perceive and react to their symptoms. Studies show that patients from families with reduced support and limited communication are more likely to delay seeking medical care, leading to diagnosis at more advanced stages of the disease [105]. Chronic stress can also affect symptom perception and care-seeking behaviors. Patients experiencing high levels of stress tend to underestimate the severity of their symptoms, leading to delays in diagnosis and treatment [106]. Reduced symptom perception in advanced stages can worsen the prognosis for patients, as they are less likely to seek timely treatment, which can lead to rapid disease progression [86].

#### 4.4.5. Integration of Neural Network Analysis for Skin Cancer Patient Profiling

The application of neural networks in this section facilitated the identification of intricate relationships between lesion visibility, inflammatory markers, and psychosocial factors. By leveraging machine learning techniques, we were able to uncover patterns that highlight how these variables interact to influence patient behavior regarding symptom recognition and medical consultation. This advanced analytical approach underscores the multifaceted nature of cancer progression and the importance of considering both biological and psychosocial elements in patient care.

In conclusion, the visibility of skin lesions and minimal inflammation in the early stages are crucial factors in the early detection of non-melanoma skin cancer. In contrast, reduced perception of symptom severity in the advanced stages, influenced by an unstable family environment and chronic stress, can delay medical consultation and worsen prognosis. Psychosocial interventions and public awareness campaigns are essential for improving symptom recognition and encouraging early medical consultation, thereby ensuring effective and timely medical intervention.

Neural network-based patient profiling was instrumental in elucidating these complex interactions, reinforcing the need for a comprehensive approach to cancer management.

### 4.5. Potential Influences of Sociodemographic and Psychological Factors on Symptom Perception and Medical Consultation in Colon Cancer

Similarly, neural networks were utilized to explore the influences of sociodemographic and psychological factors on symptom perception and medical consultation behaviors among patients with colon cancer. This methodology allowed for the identification of complex patterns and interactions that provide a deeper insight into the dynamics between biological and psychosocial factors in cancer progression.

#### 4.5.1. Inflammatory Markers and Systemic Inflammation

Patients with advanced-stage colon cancer often exhibit high levels of inflammatory markers, such as neutrophil-to-lymphocyte ratio (NLR), platelet-to-lymphocyte ratio (PLR), and the recently recognized cumulative inflammatory index (CII), especially in immunocompromised patients [107]. This chronic inflammation, exacerbated by an unstable family environment and chronic stress, can contribute to a distorted perception of symptoms. Severe systemic inflammation is associated with the release of cytokines and other pro-inflammatory molecules, which can impair normal organ function and amplify the perception of pain, discomfort, and other gastrointestinal symptoms.

#### 4.5.2. Systemic Inflammation and Colon Cancer Progression

Chronic and systemic inflammation plays a key role in the progression of colon cancer. Studies show that elevated inflammatory markers, such as NLR and PLR, are associated with poorer prognosis and reduced survival in patients with advanced colorectal cancer. High levels of these markers indicate increased tumor activity and a diminished capacity of the immune system to combat the disease [108]. The combined use of NLR and PLR has demonstrated effectiveness in diagnosing and monitoring colon cancer progression, offering superior prognostic value compared to the individual use of each marker [109].

#### 4.5.3. Distorted Symptom Perception and Care-Seeking Behaviors

Severe systemic inflammation can distort symptom perception and influence care-seeking behaviors. Patients with high levels of inflammatory markers may have a distorted perception of their symptoms, which can lead to delays in diagnosis and treatment [110]. This delay is often exacerbated by an unstable family environment and chronic stress, which can reduce patients’ attention to symptoms and prevent prompt medical consultation [111]. Neural network analysis facilitated the identification of these correlations, highlighting the significant impact of psychosocial factors on health behaviors in patients with colon cancer.

#### 4.5.4. Vagal Tone Changes and Symptom Perception

Vagal tone plays a crucial role in regulating gastrointestinal functions and the inflammatory response. Under chronic stress and inflammation, reduced vagal tone can mask symptoms or alter their perception, leading to decreased awareness of gastrointestinal symptoms. This phenomenon, known as symptom “silencing” or “masking,” can delay medical consultation because patients do not perceive the severity of their symptoms.

Studies show that chronic inflammation and stress can reduce vagal tone, thereby affecting the body’s ability to control the inflammatory response and perceive symptoms [112]. Inflammatory cytokines, such as IL-6, TNF-α, and IL-1β, can negatively influence symptom perception, contributing to the “masking” phenomenon and thus delaying early diagnosis and treatment [76]. Chronic stress exacerbates inflammation and reduces patients’ ability to perceive the severity of their symptoms, leading to delayed medical consultation and a poorer prognosis [113].

#### 4.5.5. Increased Sensitivity to Subclinical Symptoms in Early Stages

Patients with early-stage colon cancer, who exhibit lower levels of inflammation, may be more sensitive to subclinical symptoms, such as abdominal discomfort or changes in bowel habits. This increased sensitivity may result from relatively normal or elevated vagal tone, which enhances the perception of gastrointestinal signals. In these cases, a moderate inflammatory response may play a protective role, facilitating the early detection of pathological changes.

Vagal tone, through the parasympathetic nervous system, plays a crucial role in the perception of gastrointestinal signals. A normal or elevated vagal tone can enhance sensitivity to subclinical symptoms, thus facilitating the early detection of pathological changes [114]. Low levels of inflammation, reflected by markers such as NLR and PLR, contribute to a clearer and more accurate perception of gastrointestinal symptoms, enabling the early recognition of symptoms without masking clinical signs [115].

Moderate inflammation can be beneficial in the early detection of colon cancer, promoting a sufficiently thorough immune response to signal the presence of a pathological process. Studies have shown that patients with a moderate inflammatory response have a better prognosis and improved survival [116].

Patients with increased sensitivity to subclinical symptoms and normal vagal tone are more likely to seek medical care at the first signs of discomfort. This can lead to early diagnosis and more effective treatment [117].

#### 4.5.6. Integration of Neural Network Analysis for Colon Cancer Patient Profiling

The application of neural networks in this section enabled the identification of intricate relationships between inflammatory markers, sociodemographic factors, and psychological stressors. This advanced analytical approach facilitated the segmentation of patients into homogeneous subgroups based on specific combinations of factors, enhancing our ability to anticipate patient needs and implement effective, personalized interventions. Neural networks thus contributed to a more detailed understanding of how systemic inflammation and psychosocial factors influence health behaviors and cancer progression in patients with colon cancer.

In conclusion, the influences of sociodemographic and psychological factors on symptom perception and medical consultation behaviors are essential for a comprehensive understanding of colon cancer progression. Neural network analysis provided a detailed perspective on these influences, underscoring the necessity of an integrated approach that combines biological and psychosocial aspects to improve clinical outcomes and patients’ quality of life. This combined approach of traditional statistical methods and artificial intelligence demonstrates the efficacy of using neural networks in identifying risk factors and personalizing treatments for patients with colon cancer.

#### 4.5.7. Theoretical and Practical Implications

This study advances understanding by integrating sociodemographic, biological, and psychological factors through a neural network approach. Traditional statistical methods often overlook complex interactions between variables that may drive cancer progression. By using machine learning, specifically neural networks, we identify and classify patient profiles more accurately, capturing nuanced relationships. These profiles underscore the role of systemic inflammation and psychological stress in cancer progression, challenging existing models that primarily focus on single-factor relationships. This approach contributes to the theoretical framework of oncology by proposing a model where multiple dimensions (biological, psychological, and sociodemographic) interact, offering a holistic view of cancer progression. From a clinical perspective, the identified profiles suggest tailored strategies for patient care. By understanding patient groups with specific risk factors (e.g., high inflammation and stress), healthcare providers can develop personalized interventions aimed at early detection and management, potentially improving survival rates and quality of life. Moreover, this profiling approach could inform clinical guidelines, prompting regular monitoring of inflammation and stress levels in patients with high-risk cancer. Additionally, this model supports the adoption of machine learning in routine cancer diagnostics, enabling healthcare facilities to predict patient outcomes with higher accuracy and streamline decision-making processes.

### 4.6. Implications of Common Mechanisms on Therapeutic Strategies

Our comparative study of non-melanoma skin cancer (NMSC) and colorectal cancer (CRC), two cancers with differing clinical manifestations, has enabled the development of a more precise and thorough patient profiling approach. NMSC, easily detectable due to visible skin lesions, and CRC, often diagnosed at advanced stages because of nonspecific symptoms, provide an ideal framework for identifying common mechanisms and risk factors that can be extrapolated to other cancer types. This approach has significantly contributed to establishing a profiling model applicable in a broader oncological context.

In our analysis, chronic inflammation and psychosocial factors emerged as key elements in the progression of both cancer types. Elevated levels of inflammatory markers, such as the neutrophil-to-lymphocyte ratio (NLR), were associated with advanced disease stages. Additionally, factors like high-stress levels, unstable family environments, and poor living conditions negatively influenced patient outcomes. These findings align with the existing literature highlighting the role of chronic inflammation in activating NF-κB and STAT3 signaling pathways, thereby promoting cellular proliferation and tumor survival [118,119].

#### 4.6.1. Chronic Inflammation as a Therapeutic and Preventive Target

Identifying these common mechanisms offers significant opportunities for developing personalized therapeutic strategies. Anti-inflammatory therapies, such as COX-2 inhibitors, have demonstrated efficacy in reducing CRC risk by inhibiting prostaglandin E2 (PGE2) synthesis [120]. In NMSC, topical application of these inhibitors has reduced the incidence of precancerous lesions [121]. However, careful assessment of associated risks, such as cardiovascular adverse effects, is necessary when considering these therapies [122].

#### 4.6.2. Addressing Psychosocial Factors in Therapy and Prevention

Psychosocial factors play an essential role in cancer progression. Chronic psychological stress can amplify systemic inflammation and suppress antitumor immunity [123]. Psychological interventions, including mindfulness-based stress reduction techniques and cognitive-behavioral therapy, have proven effective in alleviating anxiety and depression symptoms in oncology patients [124,125]. These interventions not only enhance patients’ quality of life but may also positively impact disease progression.

#### 4.6.3. Patient Profiling for Personalized Treatment

The application of neural networks in patient profiling allowed us to identify subgroups with specific characteristics, facilitating the tailoring of therapeutic strategies to individual needs. Personalized medicine, supported by artificial intelligence, enables patient stratification based on inflammatory markers and psychosocial factors, thus guiding the selection of appropriate targeted therapies and immunotherapies [126,127].

#### 4.6.4. Integrating Sociodemographic Factors into Therapeutic Planning

Incorporating sociodemographic factors into therapeutic planning is essential for reducing disparities in oncological care. Patients from disadvantaged backgrounds or rural areas tend to present with more advanced diseases due to limited access to medical services and education [128]. Implementing adapted screening programs and health education campaigns can facilitate early detection and improve clinical outcomes [129].

#### 4.6.5. Development of a Practical Risk Assessment Tool

To facilitate the application of profiling in clinical practice, we developed a risk assessment questionnaire that enables physicians to classify patients into risk categories (low, moderate, high) based on the identified profiles. This simple and user-friendly tool supports the early identification of high-risk patients and allows for the implementation of personalized interventions.

The following table outlines the cancer risk assessment questionnaire, detailing the factors, categories, and corresponding scores (Table 10). Physicians can use this structured framework during consultations to ensure consistent and comprehensive risk evaluation.

#### 4.6.6. Instructions for Physicians:

Complete the questionnaire with the patient, assigning the corresponding score for each factor. The sum of the scores determines the patient’s total risk level, which can be interpreted using the recommendations in the table below. These guidelines offer actionable next steps tailored to the identified risk category (Table 11).

By incorporating this questionnaire into clinical workflows, physicians can efficiently identify high-risk patients, enabling timely and personalized prevention or intervention strategies. This proactive approach has the potential to significantly enhance clinical outcomes and improve the quality of life for patients.

The development of this tool was further informed by the selection of two cancer types with differing clinical manifestations. This dual focus contributed to the creation of a thorough profiling model that can be adapted and applied to other cancer contexts. The simplicity of the questionnaire ensures its practicality, while its comprehensive nature allows for a nuanced understanding of patient risk.

#### 4.6.7. Conclusions and Recommendations for Clinical Practice

The identification of chronic inflammation and psychological stress as common factors in the progression of NMSC and CRC underscores the importance of an integrated approach in cancer management. By applying patient profiling and using the risk assessment questionnaire, clinicians can offer personalized care tailored to individual needs and implement effective preventive strategies. This approach not only has the potential to improve patient prognosis and quality of life but may also serve as a model for addressing other cancer types.

While the proposed risk assessment score is derived from profiles obtained through complex neural network analyses and reflects a sophisticated integration of multifactorial data, it should be viewed as a preliminary framework. Its utility lies in offering clinicians an additional tool during consultations to identify patients at increased risk of cancer and to guide discussions around preventive measures. Given the complexity of its development, incorporating this score into clinical practice could enhance early detection efforts and contribute to personalized risk reduction strategies. However, further validation in broader and more diverse patient populations is necessary to confirm its efficacy and generalizability.

## 5. Study Limitations

One of the primary limitations of this study is its retrospective design, which inherently reduces the ability to establish clear causal relationships between the variables analyzed and the clinical outcomes observed. Additionally, the potential influence of medications administered to patients prior to hospitalization was not considered, which could impact the interpretation of inflammatory markers. Another limitation is the absence of data on the progression of inflammatory markers during hospitalization, which would have provided deeper insights into the prognosis of patients with skin carcinoma and colon cancer.

An important aspect to consider is the absence of a control group consisting of healthy individuals. While our neural network model demonstrates the ability to classify patient profiles and detect risk factors associated with non-melanoma skin cancer and colorectal cancer, including healthy individuals in this study could have enhanced the model’s applicability and robustness. However, selecting a cohort of healthy subjects presents significant challenges. It would require these individuals to come from the same environment and share similar demographic characteristics with the patient group to ensure data comparability. Such stringent selection criteria might be difficult to fulfill and could introduce additional biases.

Moreover, the selection of patients was based on specific criteria, thereby limiting the generalizability of the results to other populations or clinical settings. This study’s focus on patients from two specific clinics may introduce selection bias, further restricting the broader applicability of the findings. While the inclusion of a healthy control group is ideal, our model still provides valuable insights by identifying common mechanisms and risk factors among the patient populations studied. Future research should aim to include healthy individuals to improve the model’s generalizability and to explore potential early indicators of disease in undiagnosed individuals.

In summary, while our study offers significant findings regarding patient profiling and risk detection, these limitations highlight the need for cautious interpretation of the results and suggest directions for future research to enhance the model’s clinical relevance and applicability.

## 6. Conclusions

This study highlights significant interconnections between systemic inflammation, vagal tone, and psychosocial factors in determining the stage at which patients with non-melanoma skin cancer (NMSC) and colorectal cancer (CRC) seek medical care. Patients with low inflammation and adequate vagal tone, supported by a stable family environment, are more attuned to subclinical symptoms, enabling earlier diagnosis and timely intervention. In contrast, those with high inflammation and reduced vagal tone, often influenced by chronic stress and unstable family environments, tend to present at more advanced disease stages, leading to delayed diagnoses and accelerated progression.

A key contribution of this research is the development of a practical cancer risk assessment questionnaire that integrates clinical, sociodemographic, and psychological factors. This tool allows physicians to stratify patients into low-, moderate-, or high-risk categories, facilitating targeted preventive measures and personalized therapeutic strategies. The use of neural networks in patient profiling revealed subtle patterns and complex interactions among these factors, providing deeper insights into their influence on disease progression.

Integrating this risk assessment tool into clinical practice has the potential to transform cancer management by enhancing early detection, personalizing treatment strategies, and addressing psychosocial factors. This approach aligns with precision medicine principles, ensuring complete and patient-centered care.

Future studies should focus on validating the risk assessment tool across diverse populations and expanding its applicability to other cancer types. Additionally, exploring the underlying biological mechanisms linking systemic inflammation and psychosocial factors to cancer progression will be essential. Longitudinal research assessing the impact of personalized interventions guided by this tool on patient outcomes is also recommended.

By adopting an integrated, patient-centered approach, clinicians can better address the multifaceted needs of patients with cancer, ultimately improving clinical outcomes and enhancing quality of life.

## Figures and Tables

**Table 1:** Summary of the relevant literature on cancer incidence, progression, and risk factors.

Author (Year)	Study Focus	Risk Factors	Analytical Method	Main Findings
Siegel et al. (2023) [1]	Colorectal cancer	Global incidence	Epidemiological statistics	Cancer incidence rising, mortality declining.
Sung et al. (2021) [9]	General cancer	Global incidence	Epidemiological statistics	19.3 million cases and 10 million deaths recorded globally in 2022
Zink et al. (2018) [13]	Non-melanoma skin cancer	UV exposure, outdoor occupations	Cross-sectional study	Elevated risk of NMSC in high-UV-exposure occupations
Lutgendorf et al. (2010) [24]	Various cancers	Stress and cancer progression	Biobehavioral pathway review	Stress accelerates tumor progression via angiogenesis and invasion
Kourou et al. (2014) [29]	General applications in cancer	Prognostic ML models	Machine learning (ANN, SVM)	High predictive accuracy in cancer prognosis
Gaddis (1998) [32]	General methodology	Validation in ML studies	ANOVA, MANOVA	Importance of statistical validation for ML models
Mahamat-Saleh et al. (2019) [12]	Skin cancer	Mediterranean diet and lifestyle	Prospective cohort study	Mediterranean diet linked to reduced skin cancer risk
Lear et al. (1998) [15]	Melanoma, SCC, BCC	Skin type, UV exposure	Case-control study	Fair skin associated with increased risk across skin cancer types
Ramsay et al. (2001) [36]	Non-melanoma skin cancer	GST polymorphisms, UV exposure	Genetic analysis	GST polymorphisms linked to increased NMSC risk in transplant patients
Fryer et al. (2005) [16]	Non-melanoma skin cancer	Genetic risk, GST polymorphisms	Carcinogenesis model	Genetic predispositions increased risk in UV-exposed patients
Sawicki et al. (2021) [17]	Colorectal cancer	Genetic predispositions, family history	Epidemiological review	Family history and genetics are critical risk factors
Jimenez-Fonseca et al. (2018) [27]	Patients with cancer (general)	Anxiety, depression before treatment	Clinical study	Psychological distress worsens prognosis
Tang et al. (2022) [37]	Non-metastatic colon cancer	Real-world data application	ML-based prognostic modeling	ML models outperform traditional methods in survival prediction
Armaiz-Pena et al. (2009) [25]	Various cancers	Neuroendocrine impact on cancer progression	Pathway analysis	Neuroendocrine factors influence tumor growth
Moncada-Torres et al. (2021) [35]	Breast cancer	Survival prediction using explainable ML	ML (explainable AI, Cox regression)	Explainable AI surpasses Cox regression for survival prediction
Po-Yen Lin et al. (2022) [38]	Advanced cancer	Clinical narratives, survival prediction	Real-world clinical analysis	Clinical narratives enhance survival predictions through ML
Mathiebe et al. (2022) [39]	Skin cancer care	Healthcare needs from perspectives of patients, providers	Qualitative needs analysis	Identified gaps in healthcare service for patients with skin cancer
Nicora et al. (2020) [40]	Oncology (general)	Multi-omics analysis in cancer prediction	Multi-omics, ML integration	Multi-omics integrated with ML provides deeper insights
Buican et al. (2024) [41]	Chronic respiratory disorders	Cognitive, affective profiles, respiratory factors	Cluster analysis	Cognitive and affective factors impact respiratory disorder profiles

**Table 2:** Demographic data of this study group.

Parameter	Skin Group	Colon Group	Total	*p*
s (*n*/%)	109 (53.17%)	96 (46.83%)	205 (100%)	-
Age (years) mean ± SD	64.71 ± 15.20	66.18 ± 10.37	-	0.570 *
Gender				
Female (*n*/%)	52 (57.8%)	38 (42.2%)	90 (100%)	0.242 **
Male (*n*/%)	57 (49.6%)	58 (50.4%)	115 (100%)	
Residence				
Urban area (*n*/%)	43 (52.4%)	39 (47.6%)	82 (100%)	0.864 **
Rural area (*n*/%)	66 (53.7%)	57 (46.3%)	123 (100%)	
Smoking status				
Yes	56 (56.6%)	43 (43.4%)	99 (100%)	0.346 **
No	53 (50.0%)	53 (50.0%)	106 (100%)	
Skin				
White	71 (52.6%)	64 (47.4%)	135 (100%)	
White-Beige	33 (55.0%)	27 (45.0%)	60 (100%)	0.933 **
Beige	5 (50.0%)	5 (50.0%)	10 (100%)	
Sun exposure				
Moderate	20 (30.8%)	45 (69.2%)	65 (100%)	<0.001 ***
Intense	89 (63.6%)	51 (36.4%)	140 (100%)	
Tumor stage				
T1	41 (95.3%)	2 (4.7%)	43 (100%)	<0.001 **
T2	34 (47.9%)	37 (52.1%)	71 (100%)	
T3	25 (34.7%)	47 (65.3%)	72 (100%)	
T4	9 (47.4%)	10 (52.6%)	19 (100%)	

*p* < 0.05—statistically significant. * Man-Whitney U test. ** Chi-Square test. *** Fisher Exact test.

**Table 3:** Comparison between clinical parameters grouped by cancer type.

Parameter	Skin Cancer	Colon Cancer	*p* *
	Median (Min–Max)	Median (Min–Max)	
Leucocytes	7.20 (4.37–13.90)	7.10 (3.31–11.46)	0.768
Neutrophils	4.20 (2.12–9.00)	4.87 (1.92–8.00)	0.003 *
Lymphocytes	1.88 (0.72–3.50)	1.60 (0.40–3.02)	<0.001 *
Thrombocytes	279.00 (51–462)	227.50 (67–462)	0.001 *
Proteins	6.50 (4.40–7.80)	5.60 (2.60–7.80)	<0.001 *
RDW	13.20 (10.87–16.50)	12.80 (11.10–16.30)	0.044 *
VEM	89.70 (65.40–103.00)	87.65 (65.40–103.00)	0.002 *
NLR	2.30 (1.96–6.71)	2.68 (1.62–9.75)	0.028 *
dNLR	1.55 (0.87–5.98)	2.13 (0.94–13.21)	<0.001 *
PLR	148.7 (34.54–318.94)	140.66 (34.54–745.00)	0.202
IIC	2.75 (1.68–9.34)	3.12 (1.53–12.54)	0.001 *
MCVL	48.60 (12.39–136.22)	52.67 (32.26–182.5)	0.089

*p* < 0.05—statistically significant. * Mann–Whitney U test.

**Table 4:** Comparison of tumor stage with socio-demographic and psychological factors in patients with skin and colon cancer.

Variable	Category	Skin Cancer				Colon Cancer				*p* ^1^	*p* ^2^	*p* ^1–2^
		Stage I *n* = 41	Stage II *n* = 34	Stage III *n* = 25	Stage IV *n* = 9	Stage I *n* = 2	Stage II *n* = 37	Stage III *n* = 47	Stage IV *n* = 10			
Gender	Men	25 (61.0%)	13 (38.2%)	13 (52.0%)	9 (66.7%)	1 (50%)	24 (64.9%)	28 (59.6%)	5 (50.0%)	0.201	0.841	0.928
	Women	16 (39.0%)	21 (61.8%)	12 (48.0%)	3 (33.3%)	1 (50%)	13 (35.1%)	19 (40.4%)	5 (50.0%)			
Age		61.71 ± 17.21	64.68 ± 12.18	68.6 ± 2.66	67.67 ± 6.56	62.5 ± 12.02	70.08 ± 8.49	64.89 ± 11.00	58.5 ± 8.52	0.314	0.007 *	0.497
Environment	Urban	17 (41.5%)	15 (44.1%)	9 (36.0%)	2 (22.2%)	0 (0%)	18 (48.6%)	17 (36.2%)	4 (40.0%)	0.663	0.443	0.365
	Rural	24 (58.5%)	19 (55.9%)	16 (64.0%)	7 (77.8%)	2 (100%)	19 (51.4%)	30 (63.8%)	6 (60.0%)			
Smoking	Yes	21 (51.2%)	17 (50.0%)	13 (52.0%)	5 (55.6%)	1 (50.0%)	15 (40.5%)	23 (48.9%)	4 (40.0%)	0.993	0.876	0.957
	No	20 (48.8%)	17 (50.0%)	12 (48.0%)	4 (44.4%)	1 (50%)	22 (59.5%)	24 (51.1%)	6 (60.0%)			
Skin Type	White	25 (61.0%)	27 (79.4%)	15 (60.0%)	4 (44.4%)	0 (0.0%)	25 (67.6%)	31 (66.0%)	8 (80.0%)	0.075	0.036 *	0.807
	White-beige	15 (36.6%)	7 (20.6%)	7 (28.0%)	4 (44.4%)	1 (50.0%)	11 (29.7%)	13 (27.7%)	2 (20.0%)			
	Beige	1 (2.4%)	0 (0%)	3 (12.0%)	1 (11.1%)	1 (50.0%)	1 (2.7%)	3 (6.4%)	0 (0%)			
Sun Exposure	Moderate	6 (14.6%)	9 (26.5%)	4 (16.0%)	1 (11.1%)	1 (50.0%)	16 (43.2%)	23 (48.9%)	5 (50.0%)	0.526	0.958	0.076
	Intense	35 (85.4%)	25 (73.5%)	21 (84.0%)	8 (88.9%)	1 (50.0%)	21 (56.8%)	24 (51.1%)	5 (50.0%)			
Living Conditions	Sufficient	14 (34.1%)	10 (29.4%)	17 (68.0%)	7 (77.8%)	1 (50.0%)	18 (48.6%)	25 (53.2%)	6 (60.0%)	0.008 *	0.869	0.001 *
	Medium	14 (34.1%)	12 (35.3%)	4 (16.0%)	1 (11.1%)	1 (50.0%)	12 (32.4%)	15 (31.9%)	3 (30%)			
	Good	13 (31.7%)	12 (35.3%)	4 (16.0%)	1 (11.1%)	0 (0%)	7 (18.9%)	7 (14.9%)	1 (10.0%)			
Marital Status	Single	5 (12.2%)	7 (20.6%)	9 (36.0%)	3 (33.3%)	1 (50.0%)	10 (27.0%)	11 (23.4%)	2 (20%)	0.198	0.700	0.240
	În a relationship	3 (7.3%)	4 (11.8%)	1 (4.0%)	1 (11.1%)	0 (0%)	0 (0%)	1 (2.1%)	0 (0%)			
	Married	29 (70.7%)	21 (61.8%)	12 (48.0%)	5 (55.6%)	1 (50.0%)	25 (67.6%)	33 (70.2%)	2 (4.3%)			
	Divorced	4 (9.8%)	2 (5.9%)	3 (12.0%)	0 (0%)	0 (0%)	2 (5.4%)	2 (4.3%)	2 (20.0%)			
Family Environment	Stable	31 (75.6%)	24 (70.6%)	18 (72.0%)	2 (22.2%)	2 (100%)	28 (57.7%)	30 (63.8%)	2 (20%)	0.016 *	0.010 *	0.001 *
	Unstable	5 (12.2%)	5 (14.7%)	4 (16.0%)	3 (33.3%)	0 (0%)	5 (13.5%)	10 (21.3%)	4 (40.0%)			
	Unclear	5 (12.2%)	5 (14.7%)	3 (12.0%)	4 (44.4%)	0 (0%)	4 (10.8%)	7 (14.9%)	4 (40.0%)			
Stress Level	Low	15 (36.6%)	12 (35.3%)	5 (20.0%)	1 (11.1%)	0 (0.0%)	11 (29.7%)	7 (14.9%)	1 (10.0%)	0.007 *	0.029 *	<0.001 *
	Medium	16 (39.0%)	12 (35.3%)	5 (20.0%)	1 (11.1%)	1 (50.0%)	14 (37.8%)	12 (25.5%)	1 (10%)			
	High	10 (24.4%)	10 (29.4%)	15 (60.0%)	7 (77.8%)	1 (50.0%)	12 (32.4%)	28 (59.6%)	8 (80.0%)			
Anxiety	Rarely	10 (24.4%)	9 (26.5%)	5 (20.0%)	1 (11.1%)	2 (100%)	5 (13.5%)	6 (12.8%)	1 (10.0%)	0.667	0.040 *	0.051
	Sometimes	11 (26.8%)	10 (29.4%)	8 (32.0%)	2 (22.2%)	0 (0%)	10 (27.0%)	13 (27.7%)	2 (20.0%)			
	Often	20 (48.8%)	15 (44.1%)	12 (48.0%)	6 (66.7%)	0 (0%)	22 (59.5%)	28 (59.6%)	7 (70.0%)			
Panic	Rare	13 (31.7%)	8 (23.5%)	5 (20.0%)	1 (11.1%)	0 (0%)	7 (18.9%)	7 (14.9%)	1 (10.0%)	0.209	0.816	0.007 *
	Occasionally	13 (31.7%)	8 (23.5%)	5 (20.0%)	1 (11.1%)	1 (50.0%)	10 (27.0%)	12 (25.5%)	2 (20.0%)			
	Frequent	15 (36.6%)	18 (52.9%)	15 (60.0%)	6 (66.7%)	1 (50.0%)	20 (54.1%)	28 (59.6%)	7 (70.0%)			

* *p* < 0.05—statistically significant. *p*^1^—ANOVA test between the variable and tumor stage in skin cancer; *p*^2^—ANOVA test between the variable and tumor stage in colon cancer; *p*^1–2^—MANOVA test between the variable, tumor stage in skin cancer, and tumor stage in colon cancer.

**Table 5:** Comparison of tumor stage with biological parameters in patients with skin cancer.

Variable	Skin Cancer				Colon Cancer				*p* ^1^	*p* ^2^	*p* ^1–2^
	Stage I *n* = 41	Stage II *n* = 34	Stage III *n* = 25	Stage IV *n* = 9	Stage I *n* = 2	Stage II *n* = 37	Stage III *n* = 47	Stage IV *n* = 10			
Leucocytes	7.37 ± 2.50	6.43 ± 1.46	7.91 ± 1.28	8.40 ± 0.62	8.55 ± 1.06	6.67 ± 1.57	7.21 ± 1.25	8.02 ± 0.84	0.006 *	0.016 *	0.039 *
Neutrophils	4.44 ± 1.53	4.12 ± 1.31	5.11 ± 1.07	4.31 ± 0.11	5.22 ± 1.01	4.34 ± 1.33	5.16 ± 1.17	6.79 ± 0.84	0.041 *	<0.001 *	<0.001 *
Lymphocytes	1.83 ± 0.69	1.61 ± 0.28	1.96 ± 0.02	2.04 ± 0.03	2.60 ± 0.59	1.63 ± 0.31	1.58 ± 0.30	0.95 ± 0.37	0.012 *	<0.001 *	0.016 *
Thrombocytes	290.78 ± 107.69	253.17 ± 109.46	255.80 ± 130.34	365 ± 90.83	365.00 ± 137.17	232.24 ± 117.01	203.00 ± 68.67	231.40 ± 131.80	0.041 *	0.098	0.113
Proteins	6.22 ± 0.77	6.62 ± 0.58	6.74 ± 0.54	6.42 ± 0.42	6.50 ± 0.11	6.42 ± 0.79	4.68 ± 1.03	5.27 ± 1.12	0.008 *	<0.001 *	<0.001 *
RDW	13.59 ± 1.34	12.99 ± 1.46	13.16 ± 1.18	13.49 ± 1.00	14.20 ± 0.98	13.88 ± 1.76	12.52 ± 1.07	12.11 ± 0.48	0.244	<0.001 *	<0.001 *
MCV	87.80 ± 7.21	90.58 ± 7.76	93.48 ± 6.88	87.27 ± 3.50	92.86 ± 6.45	90.60 ± 8.15	81.03 ± 8.85	88.68 ± 8.17	0.012 *	<0.001 *	0.094
NLR	2.71 ± 1.17	3.02 ± 1.11	2.70 ± 0.53	2.15 ± 0.10	2.01 ± 0.06	3.21 ± 1.36	2.93 ± 1.29	3.56 ± 0.70	0.128	0.278	0.732
dNLR	1.59 ± 0.42	1.91 ± 0.85	2.07 ± 1.22	1.06 ± 0.12	1.56 ± 0.28	1.95 ± 0.80	3.12 ± 1.95	6.27 ± 2.81	0.005 *	<0.001 *	<0.001 *
PLR	174.56 ± 83.95	155.38 ± 56.79	130.31 ± 66.54	177.95 ± 42.77	137.95 ± 21.24	145.35 ± 71.26	139.54 ± 100.14	283.82 ± 167.99	0.077	0.001 *	0.553
IIC	3.15 ± 1.68	3.09 ± 1.39	3.20 ± 0.74	2.47 ± 0.22	2.65 ± 0.27	3.52 ± 1.79	3.65 ± 2.01	8.60 ± 2.94	0.548	<0.001 *	<0.001 *
MCVL	54.89 ± 23.43	58.76 ± 15.44	47.69 ± 3.47	42.63 ± 1.34	36.38 ± 5.82	58.75 ± 19.35	55.31 ± 24.57	104.40 ± 35.69	0.021 *	<0.001 *	0.102

* *p* < 0.05—statistically significant. *p*^1^—ANOVA test between the variable and tumor stage in skin cancer; *p*^2^—ANOVA test between the variable and tumor stage in colon cancer; *p*^1–2^—MANOVA test between the variable, tumor stage in skin cancer, and tumor stage in colon cancer.

**Table 6:** Clinical and hematological characteristics of patients with non-melanoma skin cancer according to Stable Profile, High-Risk Profile, and Advanced-Stage Profile.

Variable	Stable Profile		High-Risk Profile		Advanced Stages Profile	
	Mean ± SD	IQR (25%, 75%)	Mean ± SD	IQR (25%, 75%)	Mean ± SD	IQR (25%, 75%)
Age	64.71 ± 11.85	56.00–73.00	48.25 ± 16.80	33.00–59.25	67.83 ± 17.29	56.50–81.00
Leucocytes	6.57 ± 1.82	4.96–7.84	8.84 ± 1.69	7.63–9.80	8.01 ± 1.76	6.91–8.70
Neutrophils	3.99 ± 1.11	3.29–4.37	6.72 ± 1.59	5.91–8.00	4.75 ± 1.08	4.13–5.04
Lymphocytes	1.71 ± 0.46	1.42–1.92	1.35 ± 0.49	1.08–1.55	2.05 ± 0.38	1.93–2.05
Thrombocytes	273.66 ± 115.76	201.00–355.50	216.63 ± 27.78	191.00–232.50	293.64 ± 122.71	205.00–411.00
Proteins	6.39 ± 0.78	6.10–6.90	6.75 ± 0.22	6.57–7.00	6.56 ± 0.53	6.40–6.70
RDW	13.16 ± 1.40	12.03–14.00	13.72 ± 1.72	12.63–14.90	13.42 ± 1.15	12.62–14.14
MCV	89.73 ± 6.76	85.90–94.70	94.99 ± 3.72	94.16–97.30	89.25 ± 8.42	86.49–93.81
NLR	2.53 ± 0.58	2.17–2.62	5.38 ± 1.41	5.08–6.39	2.59 ± 0.62	2.12–2.75
PLR	165.53 ± 75.30	117.61–203.25	182.79 ± 81.11	134.20–205.00	144.55 ± 61.04	102.50–199.51
MCVL	56.02 ± 14.98	45.64–63.51	80.67 ± 34.58	62.43–88.37	44.63 ± 7.17	42.67–48.66
IIC	2.78 ± 0.52	2.47–2.97	7.01 ± 2.13	5.67–8.95	2.79 ± 0.61	2.39–3.05
DNLR	1.65 ± 0.44	1.35–2.03	3.81 ± 1.77	2.48–4.83	1.53 ± 0.40	1.26–1.72

**Table 7:** Distribution of patients with non-melanoma skin cancer according to tumor stage, family environment, living conditions, and other sociodemographic factors, based on Stable Profile, High-Risk Profile, and Advanced-Stage Profile.

Variable	Variable Type	Stable Profile Number, %	High-Risk Profile Number, %	Advanced Stages Profile Number, %
Stage	1	30 (50.85%)	3 (37.5%)	8 (19.05%)
	2	22 (37.29%)	3 (37.5%)	9 (21.43%)
	3	7 (11.86%)	2 (25.0%)	16 (38.10%)
	4	-	-	9 (21.43%)
Family Environment	Stable	55 (93.22%)	7 (87.5%)	13 (30.95%)
	Unstable	4 (6.78%)	-	13 (30.95%)
	Unclear	-	1 (12.5%)	16 (38.10%)
Living Conditions	Sufficient	13 (22.03%)	2 (25.0%)	33 (78.57%)
	Medium	22 (37.29%)	3 (37.5%)	6 (14.29%)
	Good	24 (40.68%)	3 (37.5%)	3 (7.14%)
Marital Status	Single	2 (3.39%)	1 (12.5%)	21 (50.00%)
	In a relationship	3 (5.08%)	3 (37.5%)	3 (7.14%)
	Married	50 (84.75%)	4 (50.0%)	13 (30.95%)
	Divorced	4 (6.78%)	-	5 (11.90%)
Stress Level	High	7 (11.86%)	6 (75.0%)	29 (69.05%)
	Medium	25 (42.37%)	1 (12.5%)	8 (19.05%)
	Low	27 (45.76%)	1 (12.5%)	5 (11.90%)
Anxiety	Often	20 (33.90%)	4 (50.0%)	29 (69.05%)
	Sometimes	19 (32.20%)	3 (37.5%)	9 (21.43%)
	Rarely	20 (33.90%)	1 (12.5%)	4 (9.52%)
Panic	Frequent	24 (40.68%)	5 (62.5%)	25 (59.52%)
	Occasionally	14 (23.73%)	2 (25.0%)	12 (28.57%)
	Rare	21 (35.59%)	1 (12.5%)	5 (11.90%)

**Table 8:** Clinical and hematological characteristics of patients with colon cancer according to Stable Profile, High-Risk Profile, and Advanced-Stage Profile.

Variable	Stable Profile		High-Risk Profile		Advanced Stages Profile	
	Mean ± SD	IQR (25%, 75%)	Mean ± SD	IQR (25%, 75%)	Mean ± SD	IQR (25%, 75%)
Age	69.03 ± 8.78	63.00–73.00	63.91 ± 10.63	59.50–71.50	66.94 ± 11.66	60.75–76.50
Leucocytes	6.55 ± 1.45	5.46–7.30	7.08 ± 1.24	6.27–7.85	8.43 ± 0.94	8.00–9.12
Neutrophils	4.11 ± 0.97	3.52–4.23	5.01 ± 1.07	4.02–5.78	6.95 ± 0.98	6.21–7.80
Lymphocytes	1.74 ± 0.36	1.54–1.93	1.65 ± 0.19	1.48–1.87	0.91 ± 0.31	0.70–1.07
Thrombocytes	252.88 ± 123.00	110.00–351.00	194.47 ± 73.31	117.00–231.00	230.81 ± 99.33	182.00–255.00
Proteins	6.47 ± 0.72	6.10–6.70	4.66 ± 0.99	4.10–5.20	5.71 ± 1.23	4.43–6.73
RDW	13.94 ± 1.73	12.30–15.40	12.53 ± 1.02	11.88–12.90	12.66 ± 1.48	11.72–13.05
MCV	90.16 ± 8.49	88.10–97.30	81.36 ± 8.44	75.00–87.20	89.68 ± 9.56	82.23–97.32
NLR	2.77 ± 0.97	2.17–3.11	2.73 ± 0.72	2.17–3.04	4.77 ± 1.74	3.69–5.71
PLR	145.43 ± 65.23	66.01–185.33	118.41 ± 46.71	67.93–148.18	292.93 ± 177.57	154.46–455.36
MCVL	54.25 ± 14.98	45.65–58.56	50.01 ± 8.85	41.71–57.16	109.35 ± 35.75	86.57–134.23
IIC	2.97 ± 0.65	2.47–3.21	3.15 ± 0.94	2.45–3.80	9.23 ± 2.12	8.27–11.18
DNLR	1.72 ± 0.41	1.48–2.09	2.99 ± 1.91	1.90–3.59	5.48 ± 2.55	4.29–6.25

**Table 9:** Distribution of patients with colon cancer according to tumor stage, family environment, living conditions, and other sociodemographic factors, based on Stable Profile, High-Risk Profile, and Advanced-Stage Profile.

Variable	Variable Type	Stable Profile Number, %	High-Risk Profile Number, %	Advanced Stages Profile Number, %
Stage	1	2 (6.06%)	-	-
	2	31 (93.94%)	2 (4.26%)	4 (25.0%)
	3	-	43 (91.49%)	4 (25.0%)
	4	-	2 (4.26%)	8 (50.0%)
Family Environment	Stable	27 (81.82%)	27 (57.45%)	8 (50.0%)
	Unstable	3 (9.09%)	13 (27.66%)	3 (18.75%)
	Unclear	3 (9.09%)	7 (14.89%)	5 (31.25%)
Living Conditions	Sufficient	16 (48.48%)	26 (55.32%)	8 (50.0%)
	Medium	11 (33.33%)	14 (29.79%)	6 (37.5%)
	Good	6 (18.18%)	7 (14.89%)	2 (12.5%)
Marital Status	Single	10 (30.30%)	11 (23.40%)	3 (18.75%)
	In a relationship	-	1 (2.13%)	-
	Married	22 (66.67%)	31 (65.96%)	12 (75.0%)
	Divorced	1 (3.03%)	4 (8.51%)	1 (6.25%)
Stress Level	High	10 (30.30%)	30 (63.83%)	9 (56.25%)
	Medium	13 (39.39%)	12 (25.53%)	3 (18.75%)
	Low	10 (30.30%)	5 (10.64%)	4 (25.0%)
Anxiety	Often	18 (54.55%)	30 (63.83%)	9 (56.25%)
	Sometimes	8 (24.24%)	12 (25.53%)	5 (31.25%)
	Rarely	7 (21.21%)	5 (10.64%)	2 (12.5%)
Panic	Frequent	17 (51.52%)	28 (59.57%)	11 (68.75%)
	Occasionally	10 (30.30%)	12 (25.53%)	3 (18.75%)
	Rare	6 (18.18%)	7 (14.89%)	2 (12.5%)

**Table 10:** Cancer risk assessment questionnaire.

Factor	Category	Score
Age	Under 50 years	0
	50–65 years	1
	Over 65 years	2
Perceived stress level	Low	0
	Moderate	1
	High	2
Family environment	Stable	0
	Unstable/Insecure	2
Living conditions	Adequate/Good	0
	Poor	1
Anxiety/Panic episodes	Rare/Absent	0
	Frequent	1
NLR (Neutrophil/Lymphocyte Ratio)	Below 3	0
	3–5	1
	Above 5	2
Total leukocyte count (×10³/µL)	Below 7	0
	7–9	1
	Above 9	2
Marital status	Married/In a relationship	0
	Single/Widowed/Divorced	1
Total Score		0–13

**Table 11:** Interpretation of total score.

Score Range	Risk Level	Recommendations
0–4	Low-Risk	Standard monitoring and maintenance of a healthy lifestyle. Ongoing education on self-examination and symptom awareness.
5–8	Moderate-Risk	Additional investigations, such as imaging or molecular tests. Implement interventions to reduce stress and improve family support. Lifestyle modifications, including regular physical activity and stress management techniques.
9–13	High-Risk	Detailed evaluation and referral to specialists. Immediate initiation of anti-inflammatory therapies and psychological interventions. Consultation for significant lifestyle changes, such as an anti-inflammatory diet and intensive psychological support programs.

## Data Availability

The authors declare that the data of this research are available from the corresponding authors upon reasonable request.
